# Supplementary material for: Robust single-cell matching and multimodal analysis using shared and distinct features
Source: Nat Methods. 2023 Jan 9;20(2):304–15. doi: 10.1038/s41592-022-01709-7 (PMC9911356; doi:10.1038/s41592-022-01709-7)
Supplement: Supplementary file 1 — Supplementary Figs. 1–23, Methods and Materials. [file 41592_2022_1709_MOESM1_ESM.pdf]

nature methods

Article

https://doi.org/10.1038/s41592-022-01709-7

Robust single-cell matching and multimodal analysis using shared and distinct features

In the format provided by the authors and unedited

Supplementary Notes for : Robust Single-cell Matching and Multi-modal Analysis Using Shared and Distinct Features

Bokai Zhu[1,2,*], Shuxiao Chen[3,*], Yunhao Bai[2,4], Han Chen[2], Guanrui Liao[5], Nilanjan Mukherjee[2], Gustavo Vazquez[2], David R McIlwain[2], Alexandar Tzankov[6], Ivan T Lee[2], Matthias S Matter[6], Yury Goltsev[2], Zongming Ma[3,†], Garry P Nolan[2,†], and Sizun Jiang[5,7,8,†]

[1]Department of Microbiology and Immunology, Stanford University, Stanford, CA, United States
[2]Department of Pathology, Stanford University, Stanford, CA, United States
[3]Department of Statistics and Data Science, The Wharton School, University of Pennsylvania, PA, United States
[4]Department of Chemistry, Stanford University, Stanford, CA, United States
[5]Center for Virology and Vaccine Research, Beth Israel Deaconess Medical Center, Boston, MA, United States
[6]Pathology, Institute of Medical Genetics and Pathology, University Hospital Basel, University of Basel, Basel, Switzerland
[7]Department of Pathology, Dana Farber Cancer Institute, Boston, MA, United States
[8]Broad Institute of Harvard and MIT, Cambridge, MA, United States
[†]These authors jointly supervised this work
[*]These authors contributed equally

Correspondence: ***zongming@wharton.upenn.edu, gnolan@stanford.edu, sjiang3@bidmc.harvard.edu***

Supplementary Figures

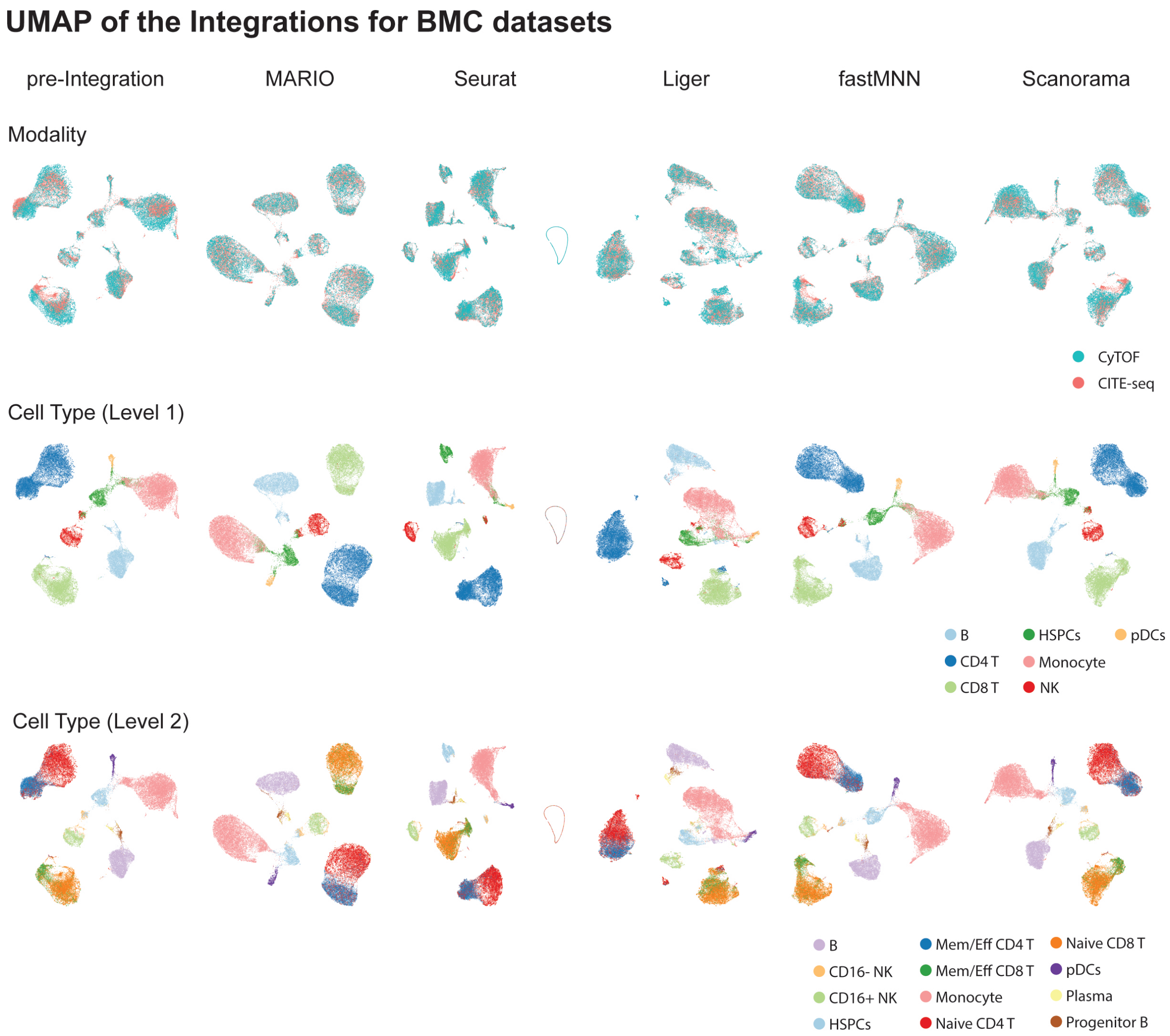

Supplementary Fig 1: Performance of matching and integration on cross-modality CyTOF and CITE-seq bone marrow cells. UMAP plots visualizing pre-integration and post-integration results with different methods. For methods other than MARIO, only shared features were used during integration, colored by modality or cell type annotation (level 1 and level2).

t-SNE Plots During Feature Dropping of BMC dataset (Alphabetical)

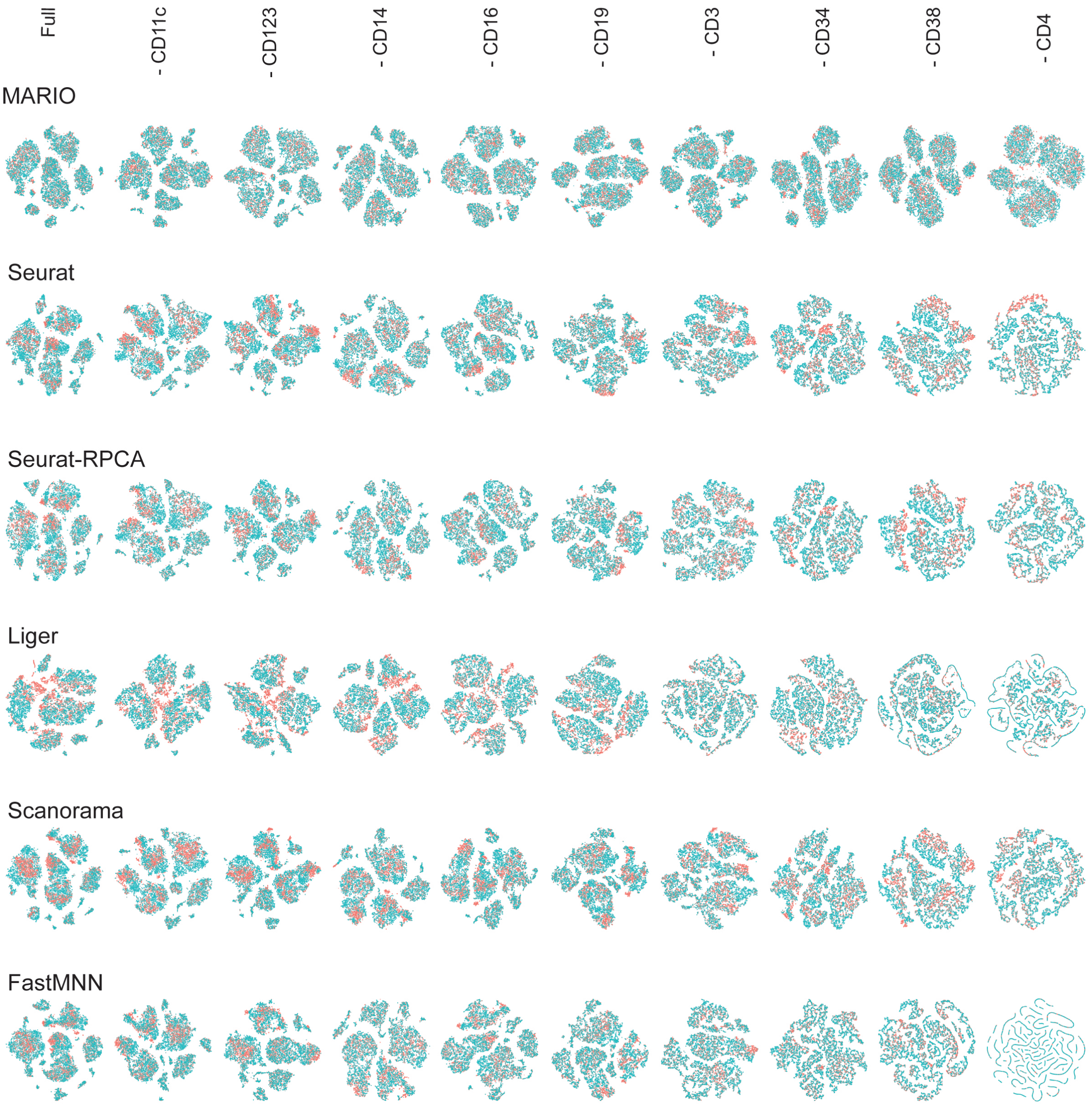

Supplementary Fig 2: Performance of matching and integration on cross-modality CyTOF and CITE-seq bone marrow cells. t-SNE plots visualizing post-integration results with different methods, during each sequential protein feature drop step (alphabetic).

Sequentially Deleting Overlapping Protein Features by Importance

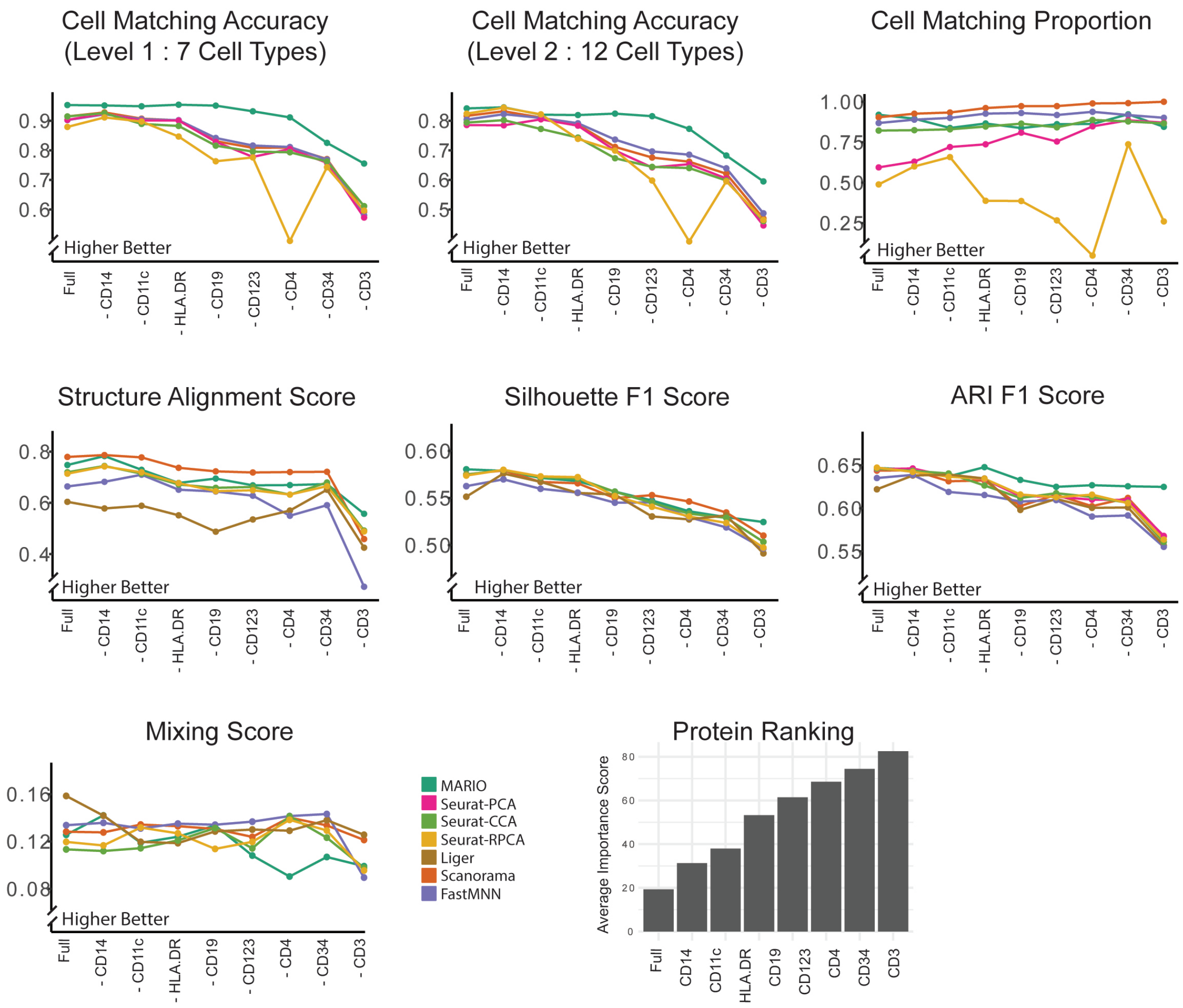

Supplementary Fig 3: Sequentially dropping shared protein features by importance. Same analysis performed in Extended Data Fig 2 but dropping shared features by importance score (less important dropping first). Importance scores were calculated from a permutation feature importance test with a Random Forest model predicting cell type level 2 from shared protein features.

t-SNE Plots During Feature Dropping of BMC dataset (Importance Score)

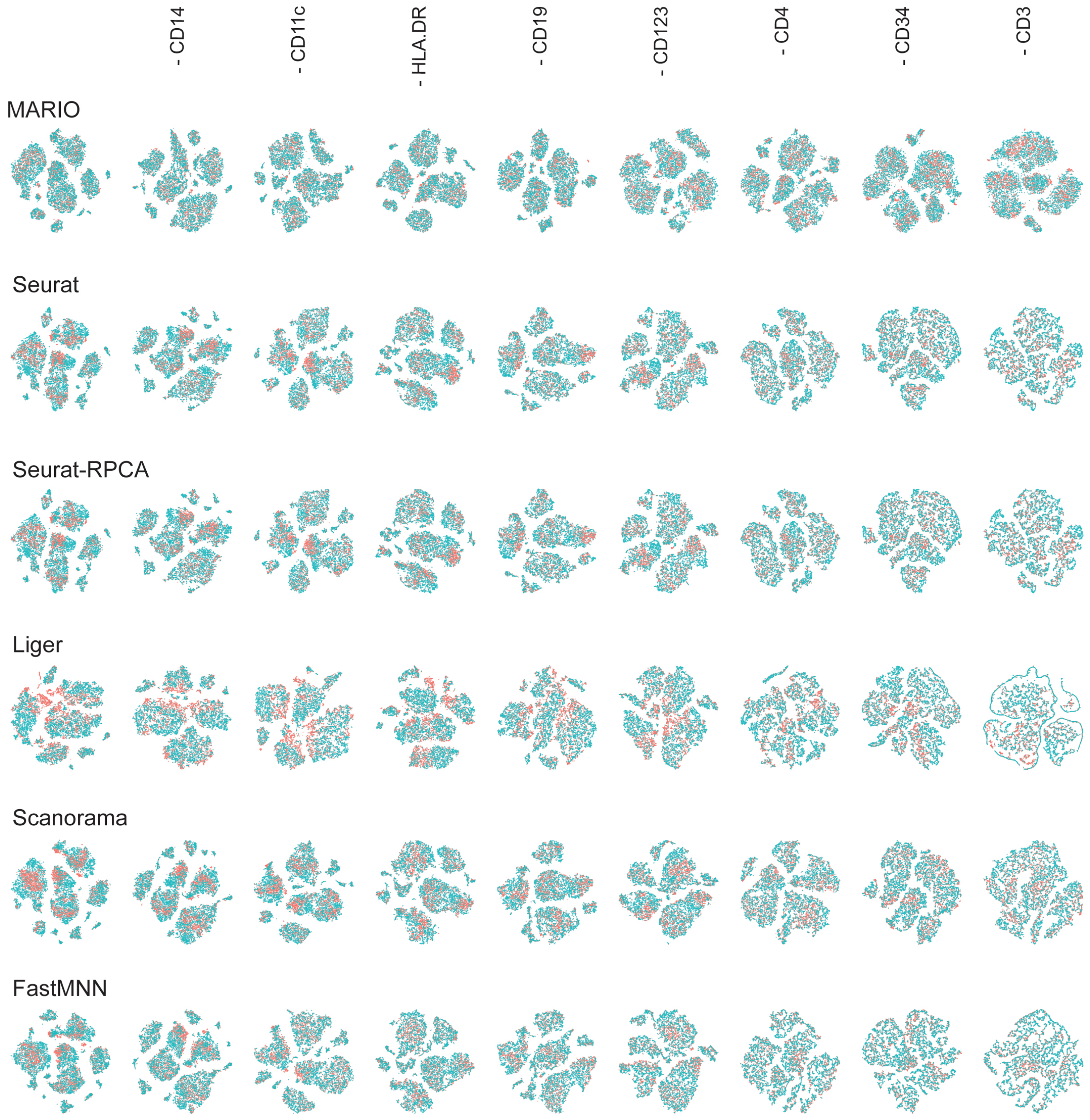

Supplementary Fig 4: Sequentially dropping shared protein features by importance. t-SNE plots visualizing post-integration results with different methods, during each sequential protein feature drop step (importance).

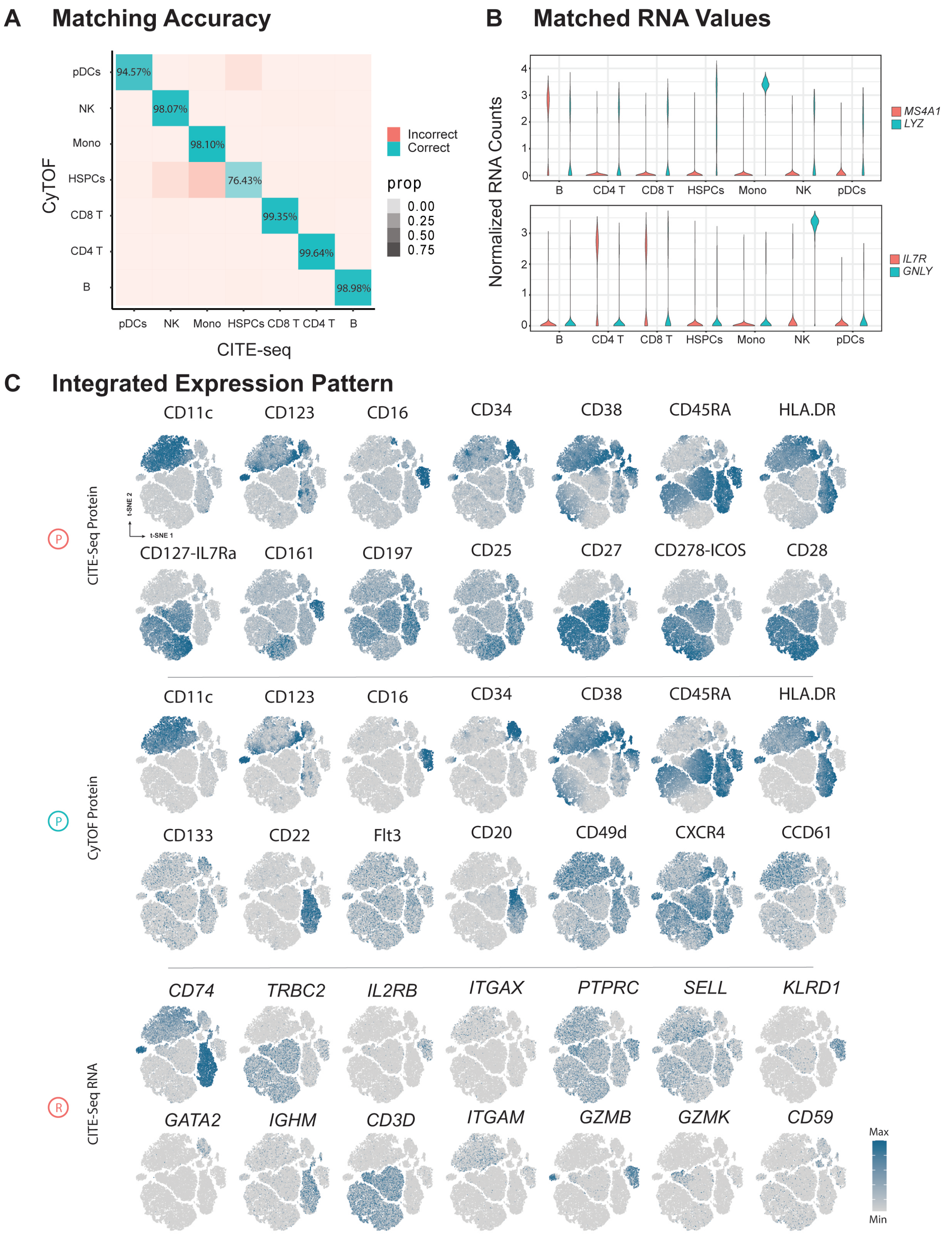

Supplementary Fig 5: Matching and integration of cross-modality CyTOF and CITE-seq bone marrow data with MARIO. (A) Confusion matrix with MARIO cell-cell matching accuracy (balanced accuracy) across cell types. **(B)** Violin plots of normalized RNA counts among different MARIO matched CITE-seq and CyTOF cell types. **(C)** t-SNE plots of the matched cells with protein/RNA expression levels overlaid as an extension of Figure 2G.

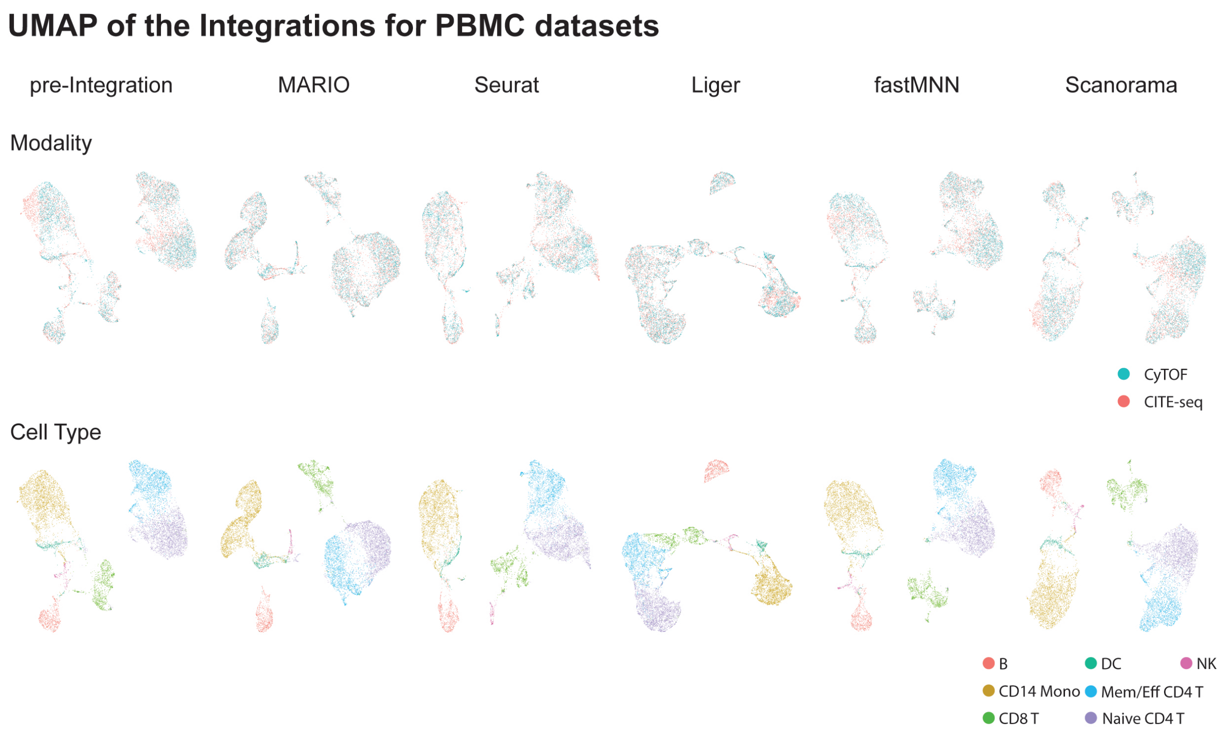

Supplementary Fig 6: Performance of matching and integration on cross-modality CyTOF and CITE-seq PBMCs. Umap plots visualizing pre-integation and post-integration results with different methods.

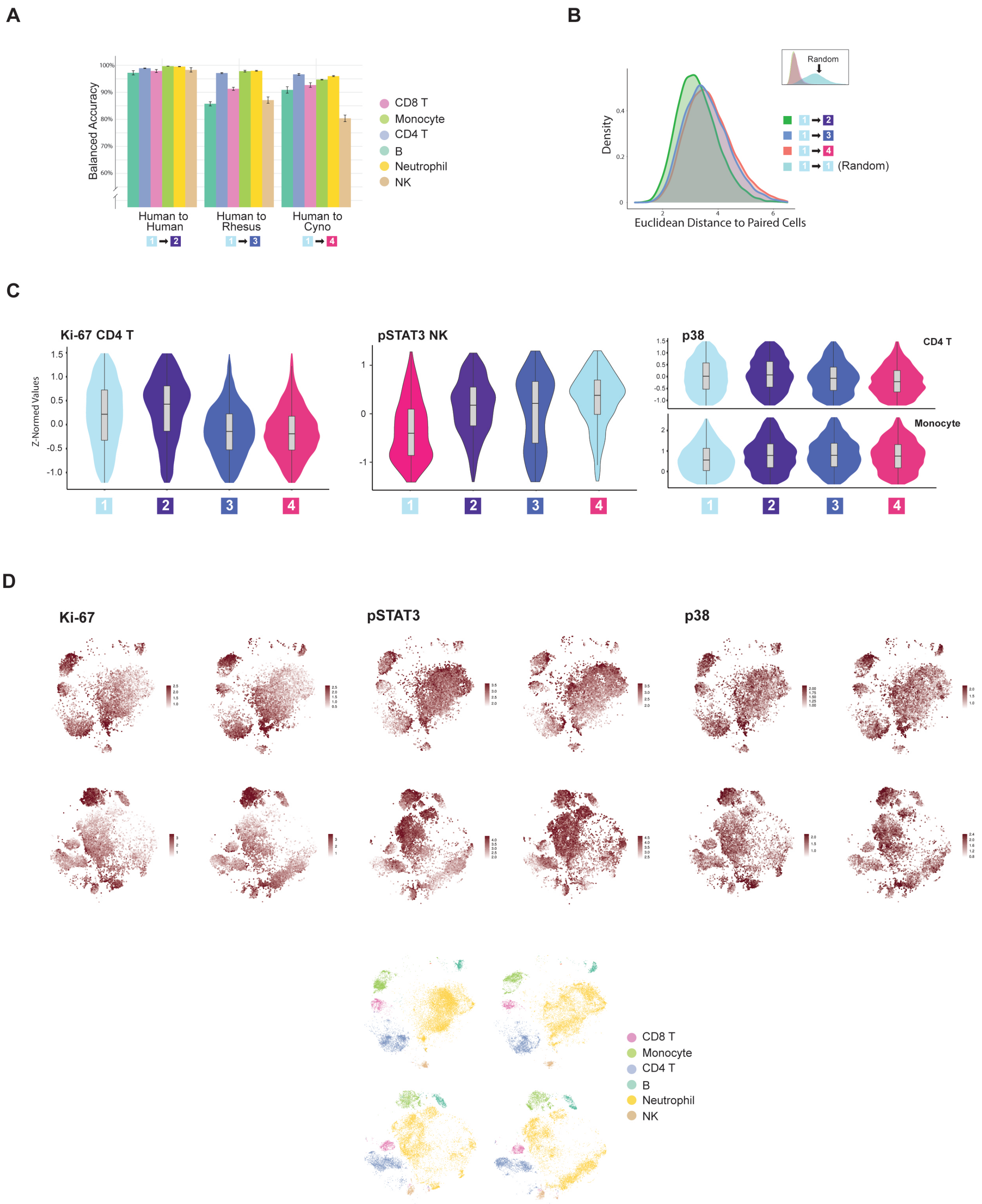

Supplementary Fig 7: Matching and integration on cross-species whole blood cells CyTOF data (H1N1 and IFN-gamma). (A) Balanced accuracy (mean ± sd, n = 5) for each cell type after MARIO matching, for cells from Rahil et al. to other datasets. **(B)** Euclidean distance of canonical correlations for pairs of matched versus random cells between Rahil et al. to other datasets. **(C)** Violin plot (5% - 95% quantile) of the normalized expression levels of Ki-67, pSTAT3 and p38 across the four datasets for the specified cell types: CD4 T cells and monocytes. **(D)** t-SNE plots of pre-integration data, with expression levels of Ki-67, pSTAT3 and p38 across the four datasets.

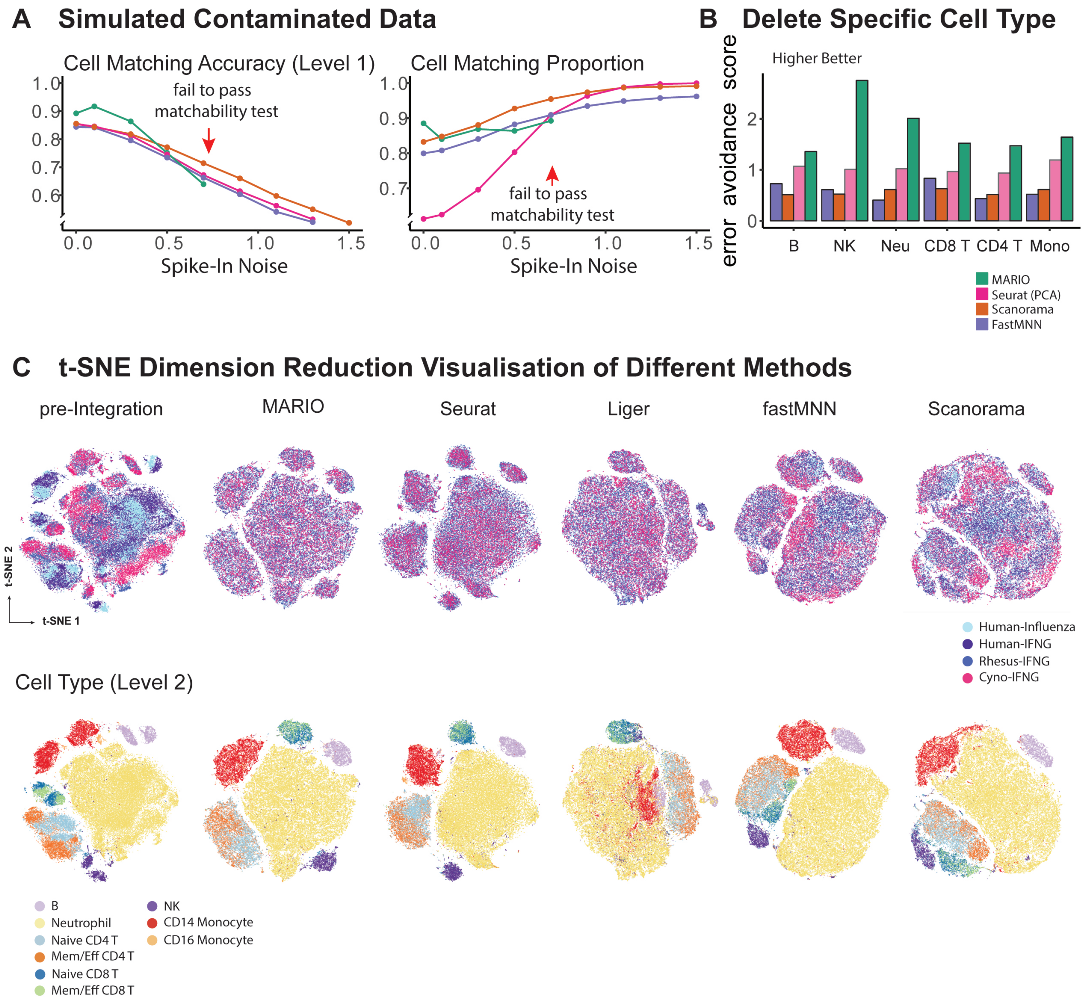

Supplementary Fig 8: Performance of matching and integration on cross-species whole blood cells CyTOF data (H1N1 and IFN-gamma). (A) Testing algorithm stringency between different methods. Increasing amounts of random spike-in noise was added to the data, and the matching accuracy and proportion of cells matched to X were quantified. MARIO matchability test automatically suspended forced matching of inappropriate data due to poor quality here. **(B)** Testing algorithm stringency among different methods. Single-cell types in Y were deleted before matching to X. The proportion of cells belonging to the deleted cell type in matched X cells were used to calculate the erroneous avoidance score. **(C)** t-SNE plots visualizing pre-integation and post-integration results with different methods.

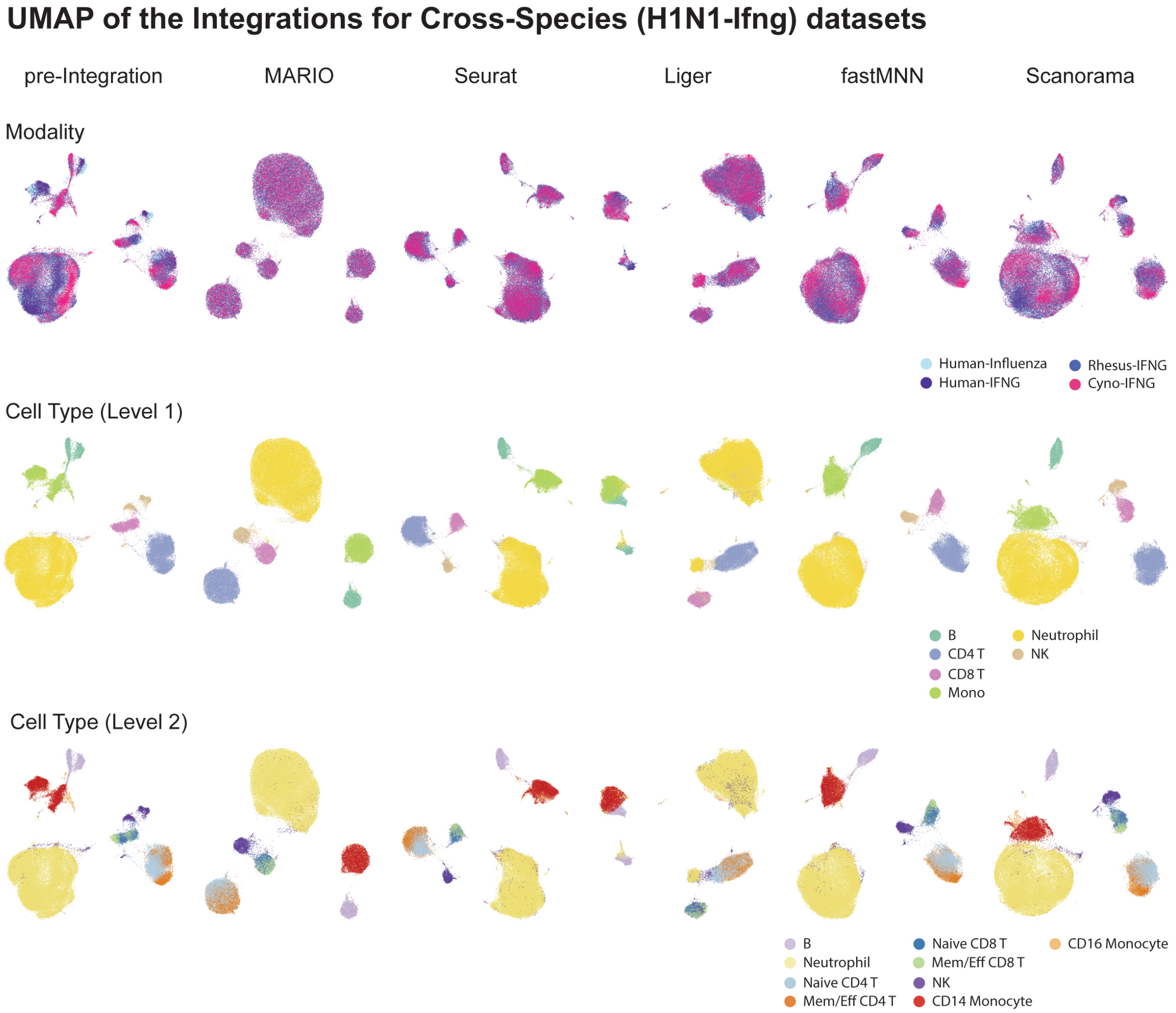

Supplementary Fig 9: Performance of matching and integration on cross-species whole blood cells CyTOF data (H1N1 and IFN-gamma). UMAP plots visualizing pre-integration and post-integration results with different methods.

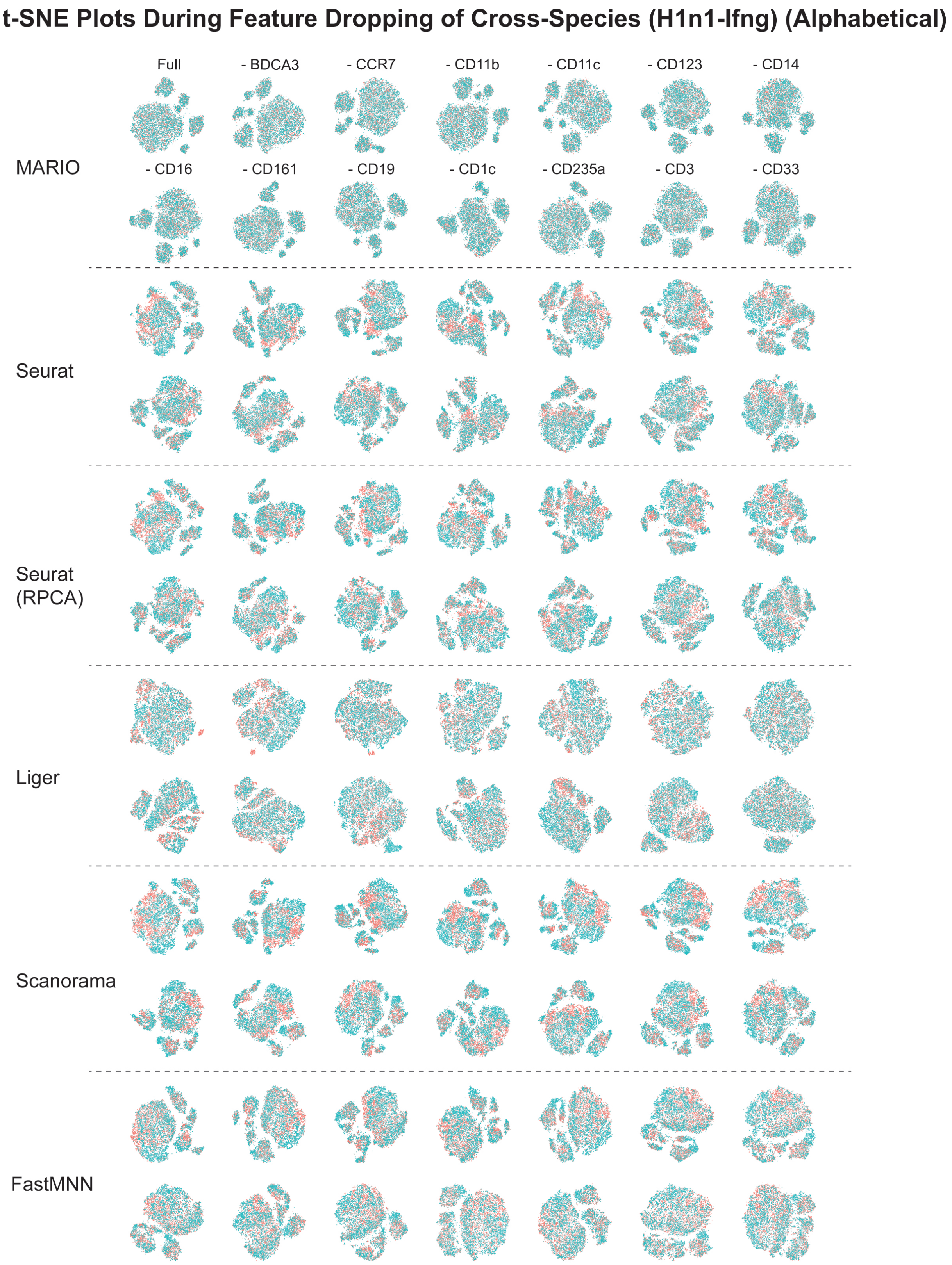

Supplementary Fig 10: Performance of matching and integration on cross-species whole blood cells CyTOF data (H1N1 and IFN-gamma). t-SNE plots visualizing post-integration results with different methods, during each sequential protein feature drop (alphabetical).

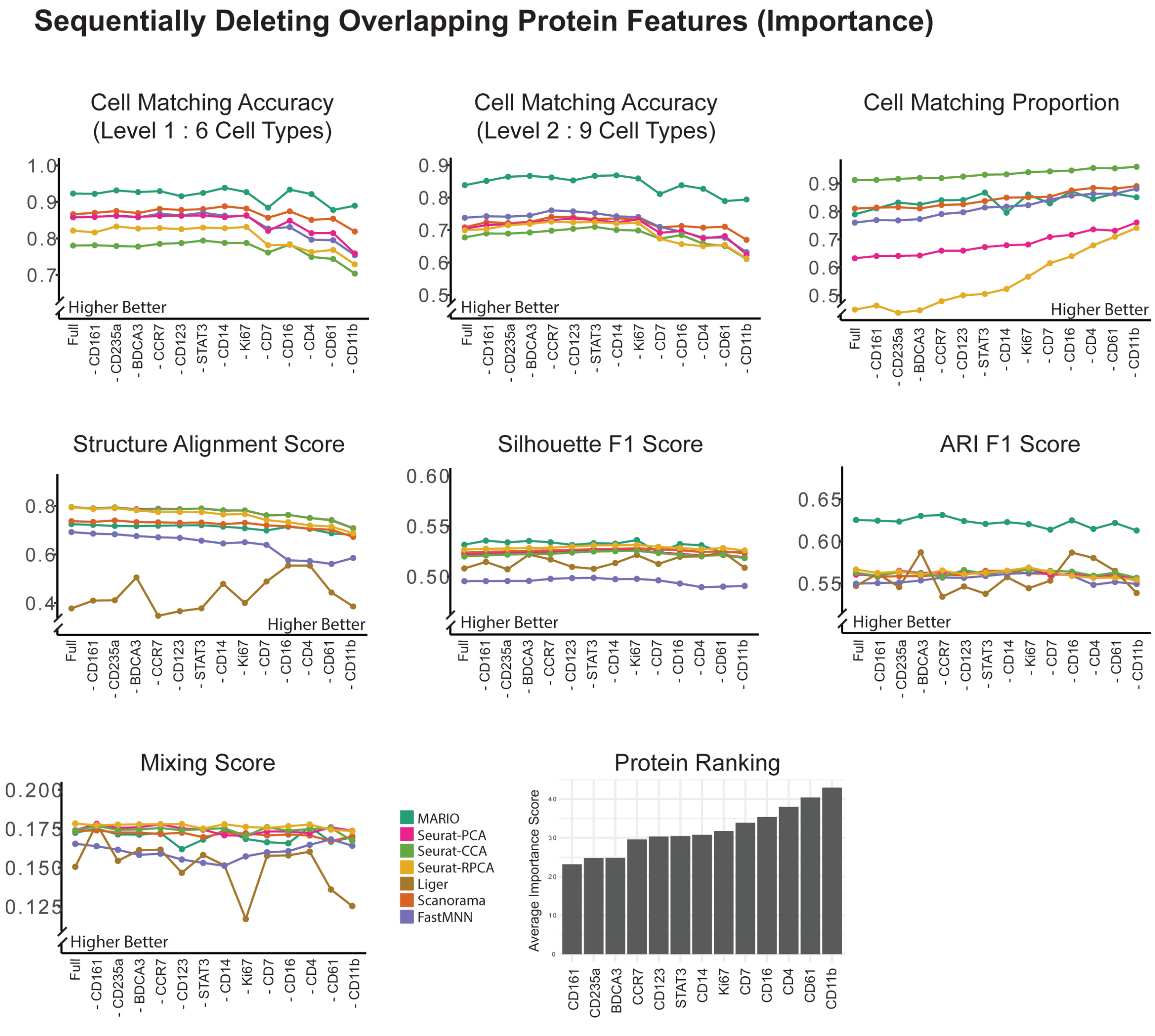

Supplementary Fig 11: Sequentially dropping shared protein features by importance. Same analysis performed in Extended Data Fig 5 but dropping shared features by importance score (less important dropping first). Importance scores were calculated from a permutation feature importance test with a Random Forest model predicting cell type level 2 from shared protein features.

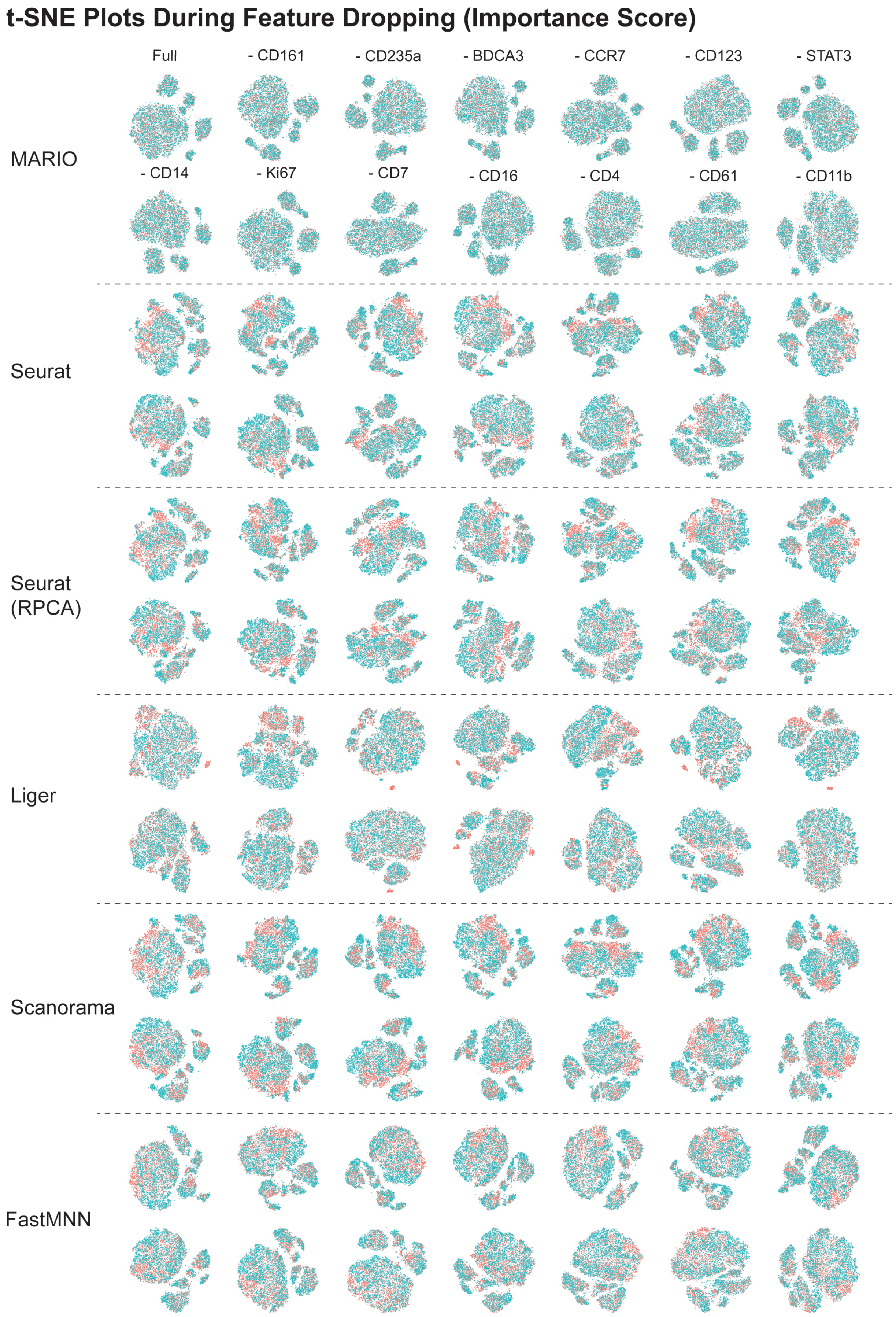

Supplementary Fig 12: Sequentially dropping shared protein features by importance. t-SNE plots visualizing post-integration results with different methods, during each sequential protein feature drop step (importance).

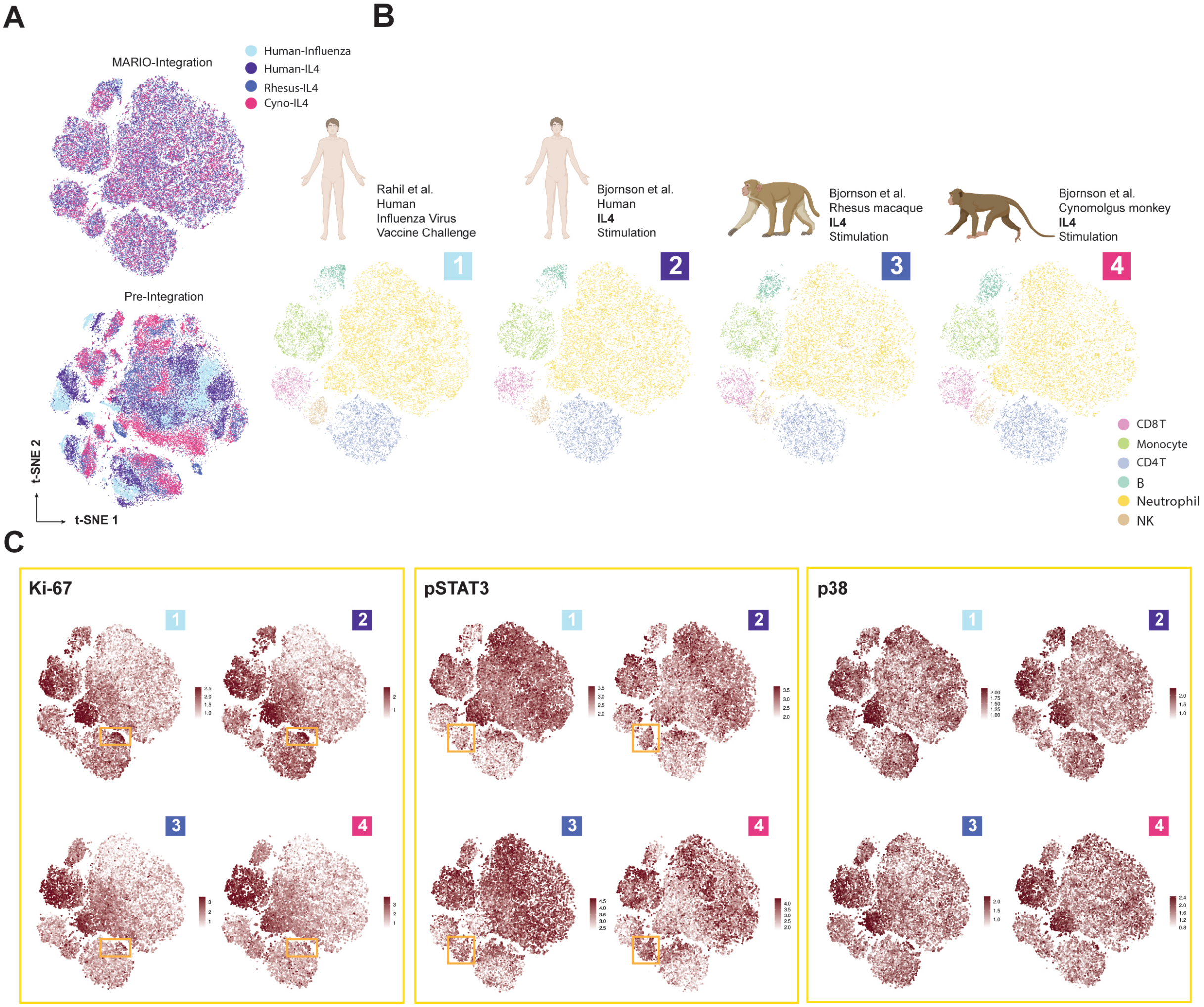

Supplementary Fig 13: Cross-species H1N1 Challenge and IL-4 integrative analysis with MARIO. MARIO integration of human, rhesus macaque and cynomolgus monkey whole blood cells from a H1N1 challenge study or IL-4 stimulation. **(A)** t-SNE plots of the four datasets, pre-integration and post MARIO-integration as colored by dataset of origin. **(B)** t-SNE plots of each individual dataset, colored by cell type annotation. **(C)** t-SNE plots with expression levels of Ki-67, pSTAT3 and p38 across four datasets.

Sequentially Deleting Overlapping Protein Features (Alphabetical)

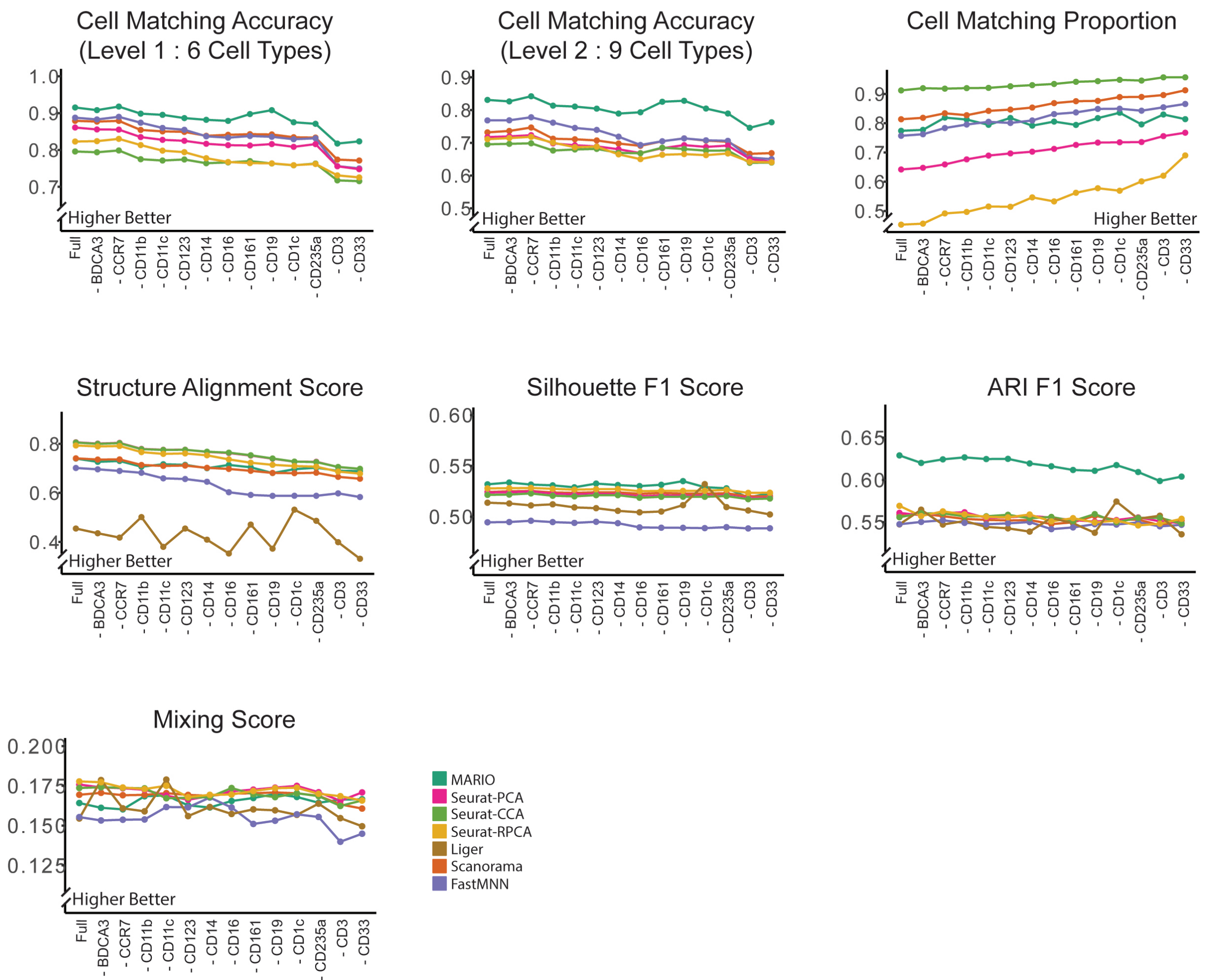

Supplementary Fig 14: Performance of matching and integration on cross-species whole blood cells CyTOF data (H1N1/IL-4 group). Performance of matching and integration during sequentially dropping of shared protein features. The tested parameters are: cell-cell matching accuracy, proportion of cell in X matched, average Structure alignment score, Silhouette F1 score, Adjusted Rand Index F1 score and average Mixing score.

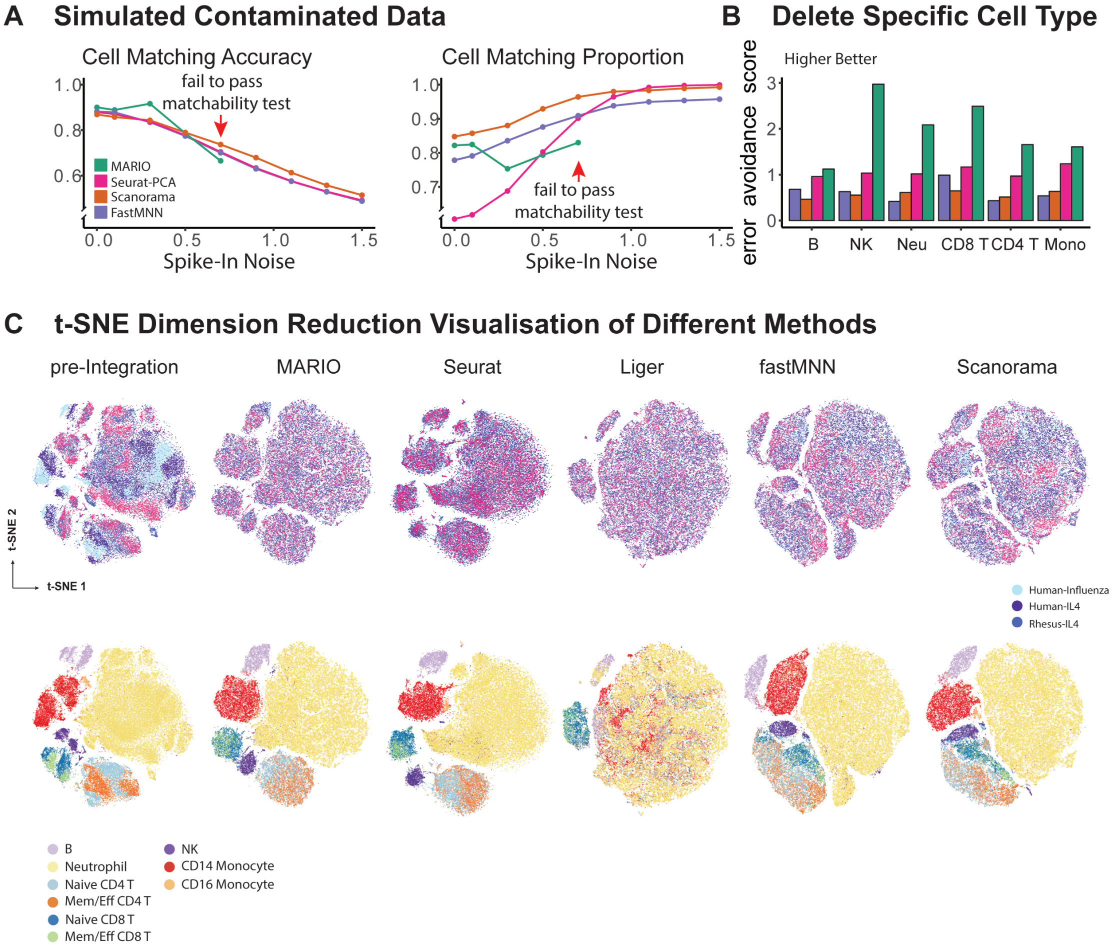

Supplementary Fig 15: Performance of matching and integration on cross-species whole blood cells CyTOF data (H1N1/IL-4 group). (A) Testing algorithm stringency between different methods. Increasing amounts of random spike-in noise was added to the data, and the matching accuracy and proportion of cells matched to X were quantified. MARIO matchability test automatically suspended forced matching of inappropriate data due to poor quality here. **(B)** Testing algorithm stringency among different methods. Single-cell types in Y were deleted before matching to X. The proportion of cells belonging to the deleted cell type in matched X cells were used to calculate the erroneous avoidance score. **(C)** t-SNE plots visualizing pre-integation and post-integration results with different methods.

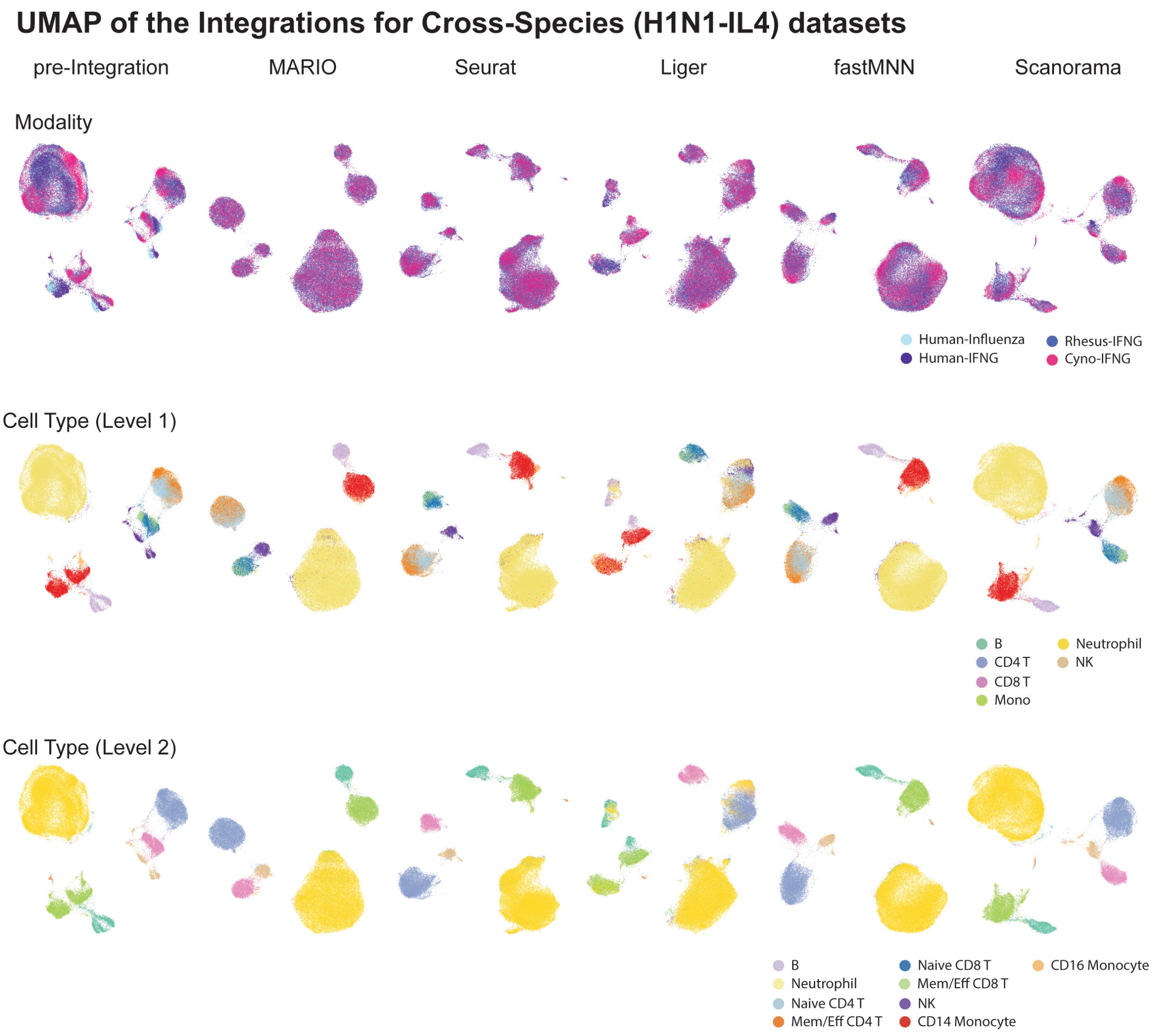

Supplementary Fig 16: Performance of matching and integration on cross-species whole blood cells CyTOF data (H1N1/IL-4 group). UMAP plots visualizing pre-integration and post-integration results with different methods.

UMAP of the Integrations for Murine Spleen datasets

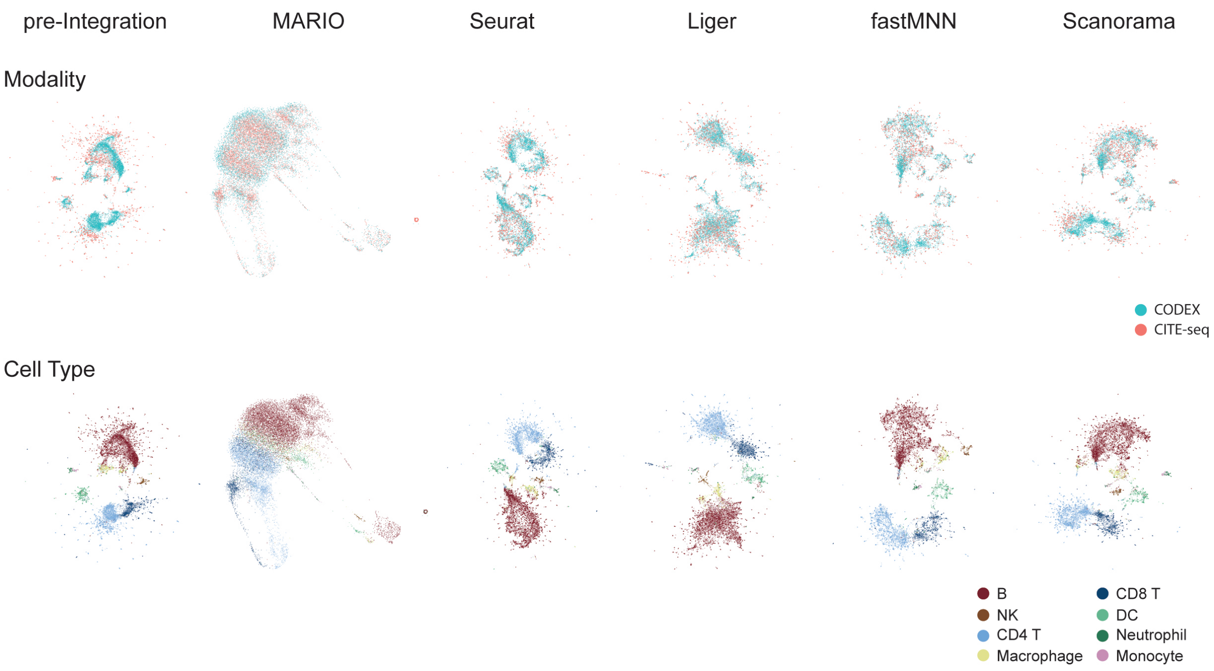

Supplementary Fig 17: Performance of matching and integration on CODEX and CITE-seq murine spleen cells. t-SNE plots visualizing pre-integation and post-integration results with different methods.

Sequentially Deleting Overlapping Protein Features (Importance)

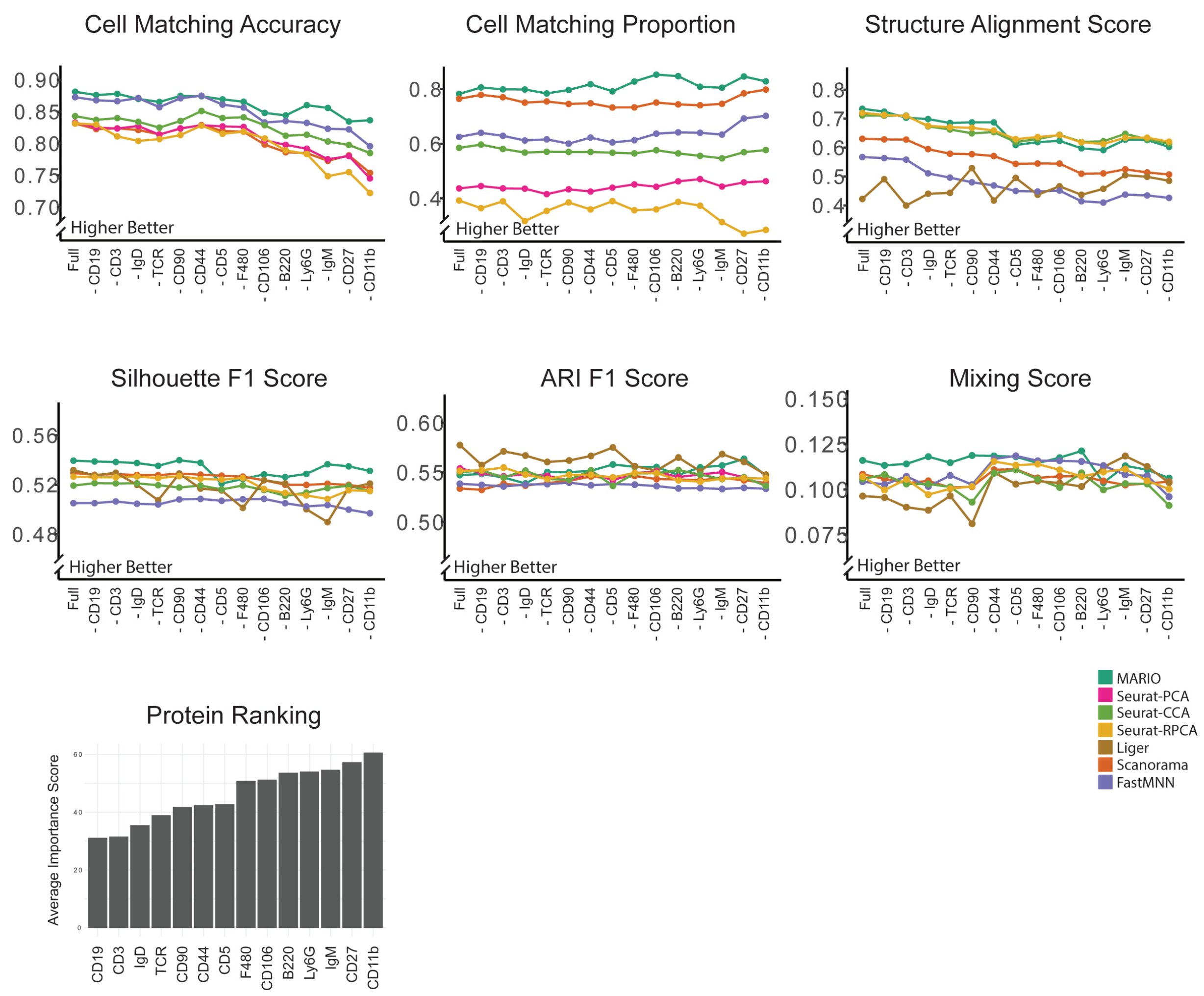

Supplementary Fig 18: Sequentially dropping shared protein features by importance. Same analysis performed in Extended Data Fig 9 but dropping shared features by importance score (less important dropping first). Importance scores were calculated from a permutation feature importance test with a Random Forest model predicting cell type level 2 from shared protein features.

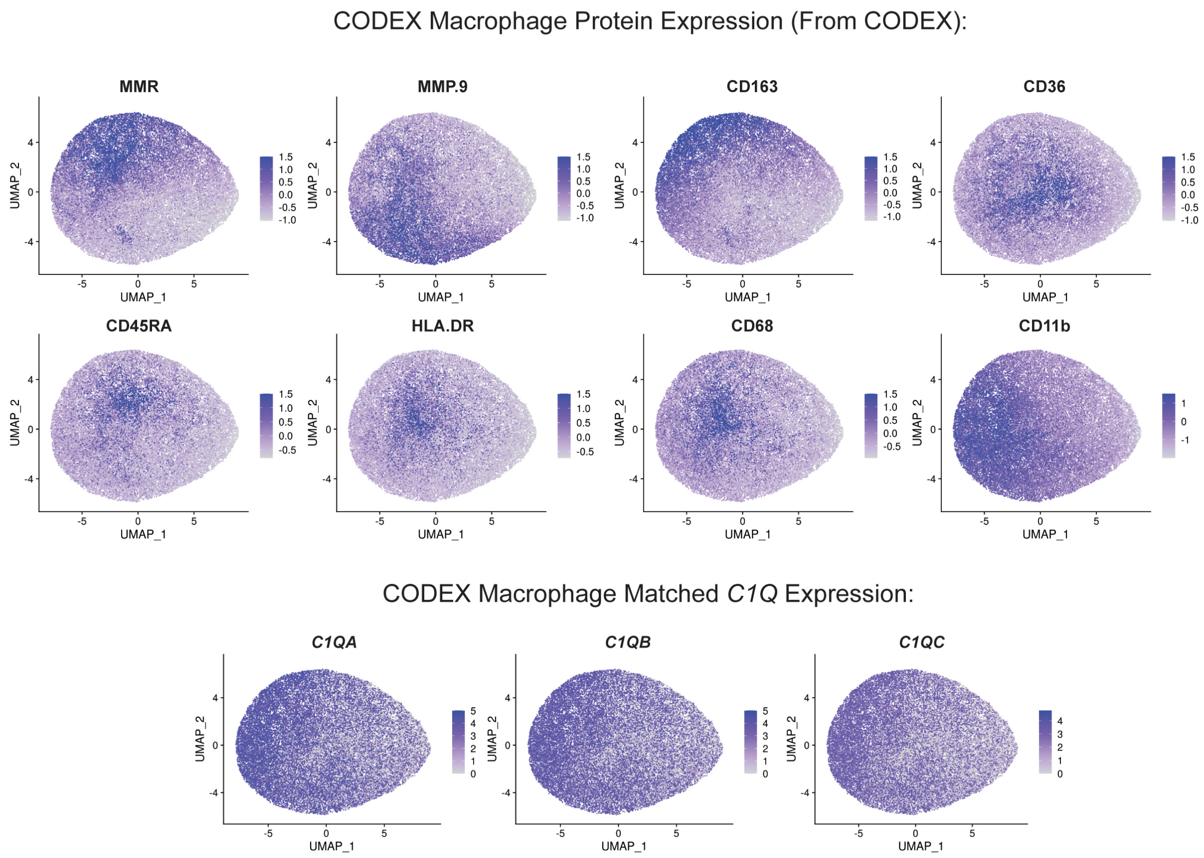

Supplementary Fig 19: MARIO analysis on COVID-19 lung tissue and BALF cells. UMAP plots of matched CODEX macrophage cells (reduction calculated with only CODEX proteins), overlaid with CODEX macrophage related protein markers, and matched CITE-seq RNA counts.

Benchmarking Parameters of MARIO on BMC-CITE-seq/CyTOF matching

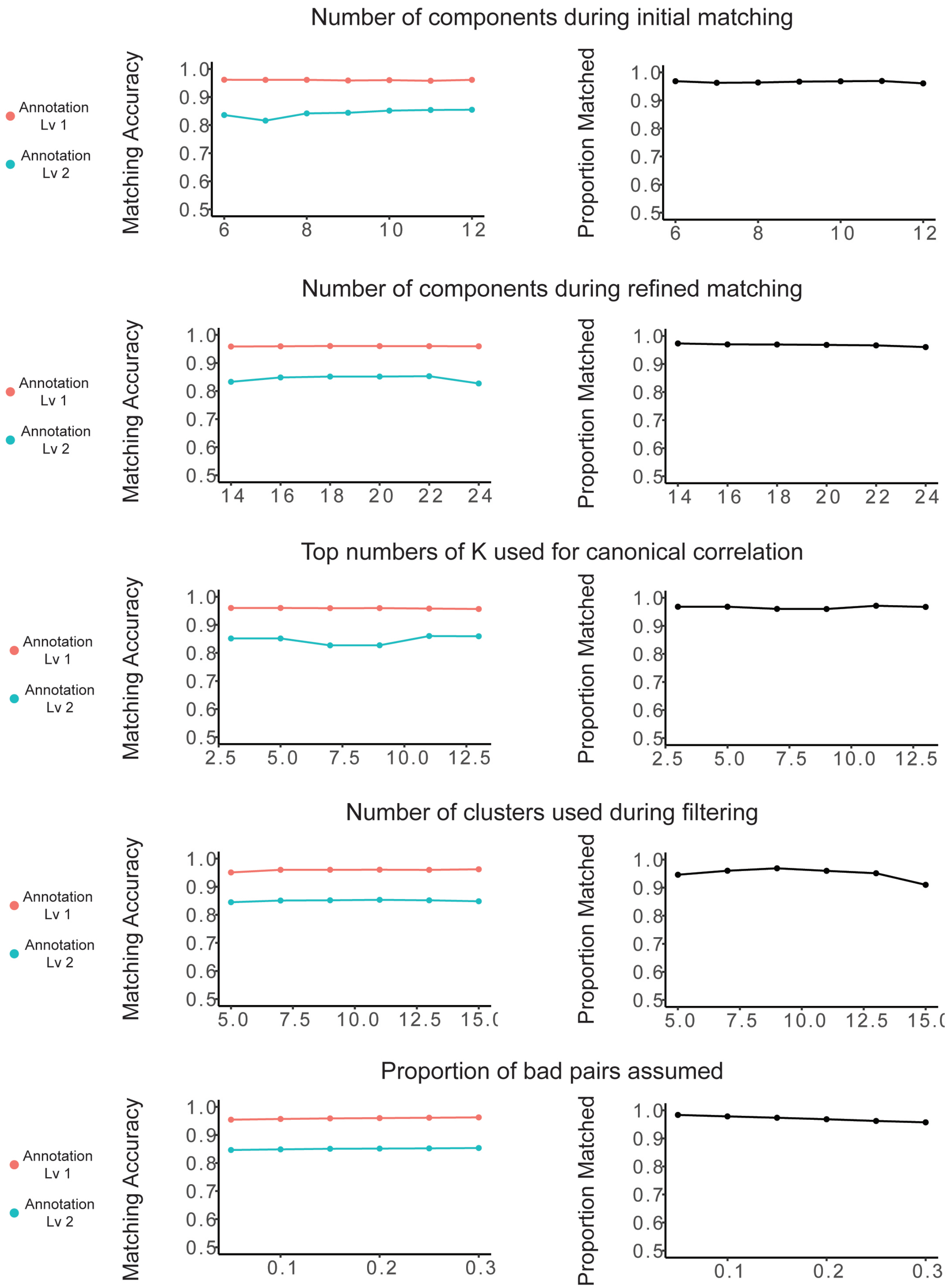

Supplementary Fig 20: MARIO parameter benchmarking Run MARIO parameters (numbers of components used during initial matching; number of components used during refined matching; top number of K used for canonical correlation; numbers of clusters used during filtering; proportion of bad pairs assumed during filtering) benchmarked on the Bone Marrow dataset (dataset used in Figure 2).

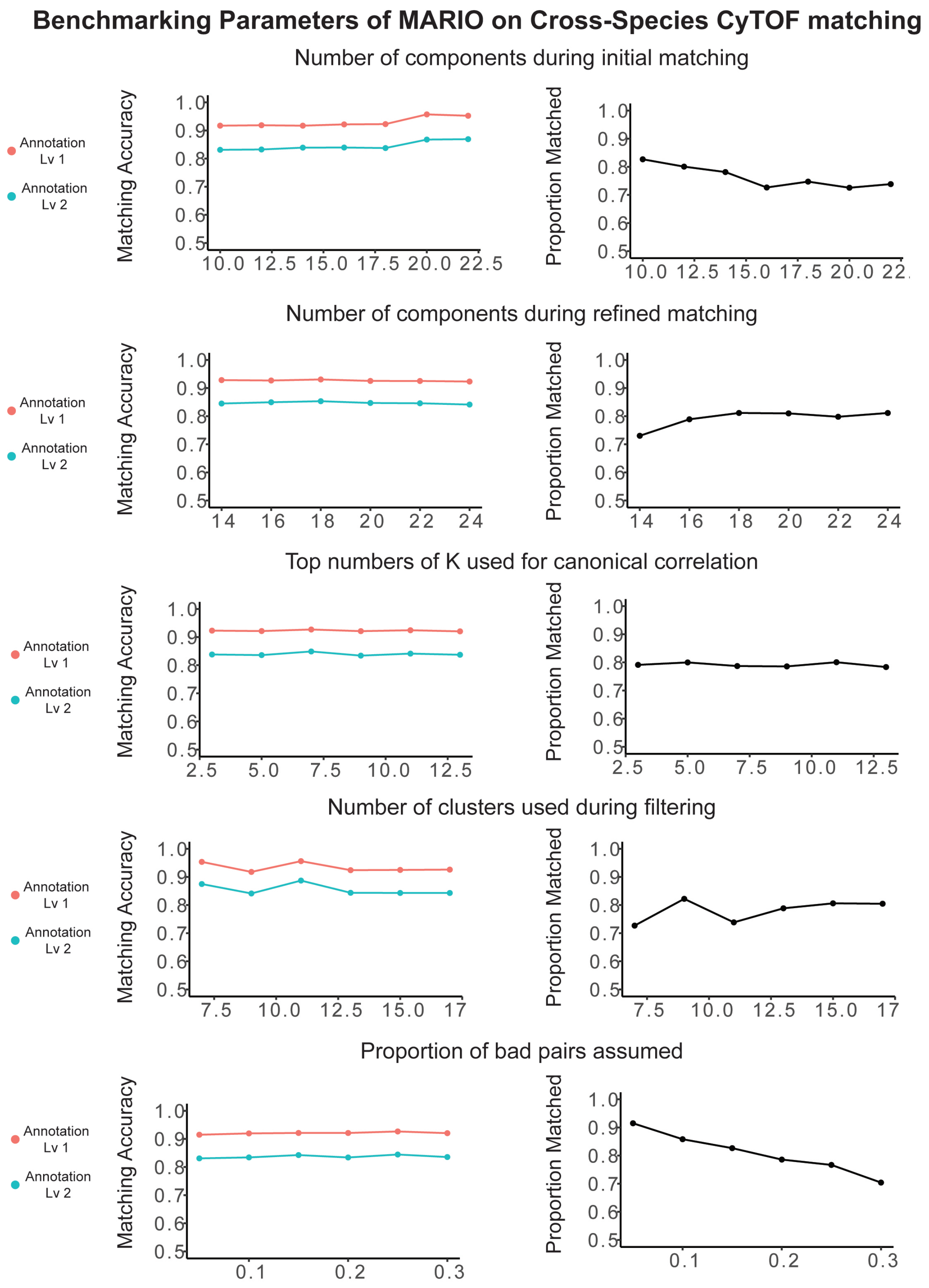

Supplementary Fig 21: MARIO parameter benchmarking Run MARIO parameters (numbers of components used during initial matching; number of components used during refined matching; top number of K used for canonical correlation; numbers of clusters used during filtering; proportion of bad pairs assumed during filtering) benchmarked on the Cross-species dataset (dataset used in Figure 3).

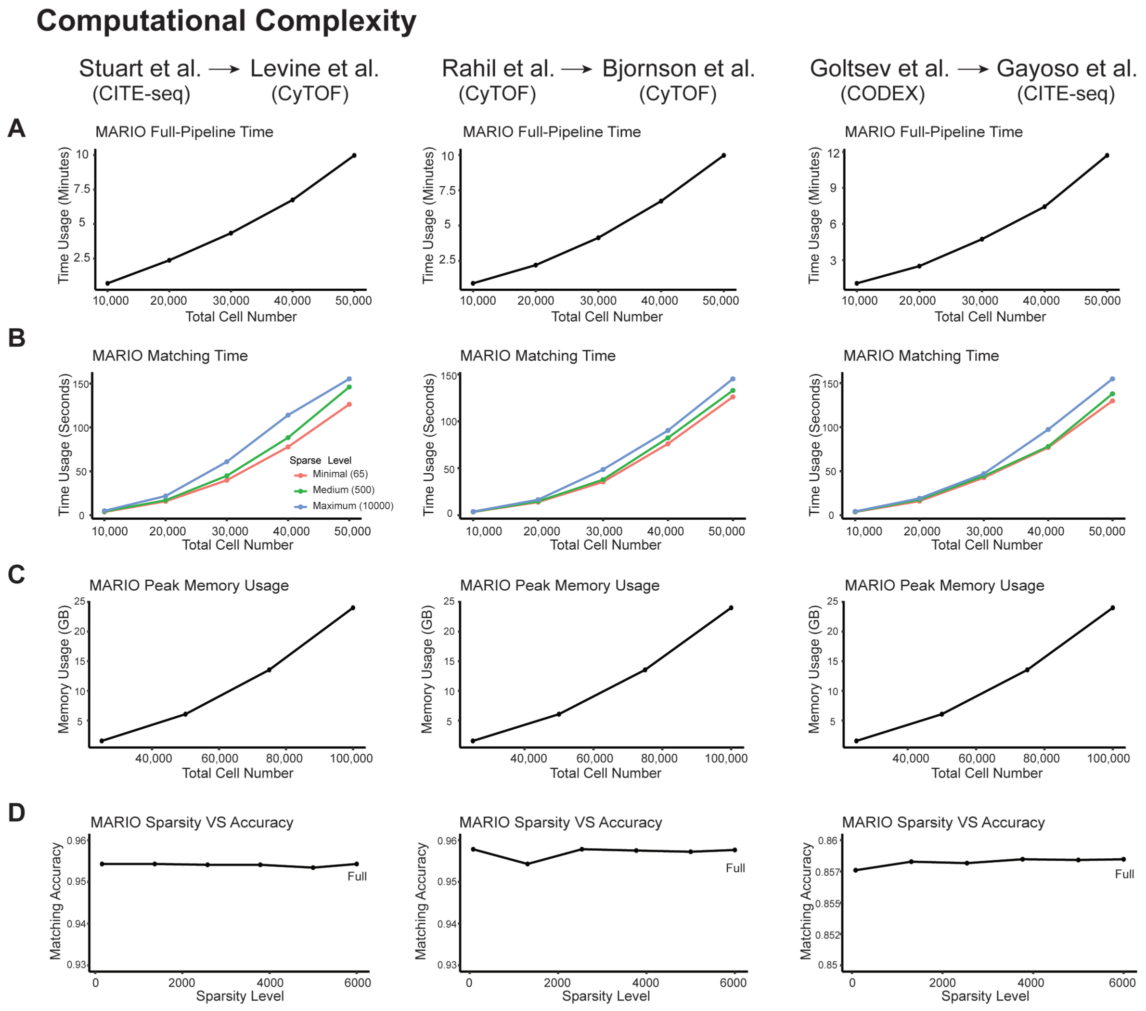

Supplementary Fig 22: Computational complexity (A) Run time for full MARIO pipeline (Initial and refined matching; Finding the best interpolation; Joint regularized filtering; CCA calculation) across different datasets. **(B)** Run time for MARIO matching steps (total time for initial and refined matchings) across different datasets. The ratio of X and Y was set as 1:4 (eg. at a total of 20,000 cells, X has 4000 cells and Y has 16,000 cells). Three sparsity levels were shown in the figures, which are 1: 'Minimal' sparsity calculated by MARIO. 2: 'Maximum' sparsity, same as using dense data. 3: 'Medium' sparsity which is the level in the middle between minimal and maximum. **(C)** Peak memory usage when running the full MARIO pipeline across different datasets. The ratio of X and Y was set as 1:4. **(D)** Matching accuracy with different levels of sparsity for MARIO. Total of 50,000 cells were used, where the ratio of X and Y was set as 1:4.

MARIO Compared to Optimal Transport Matching

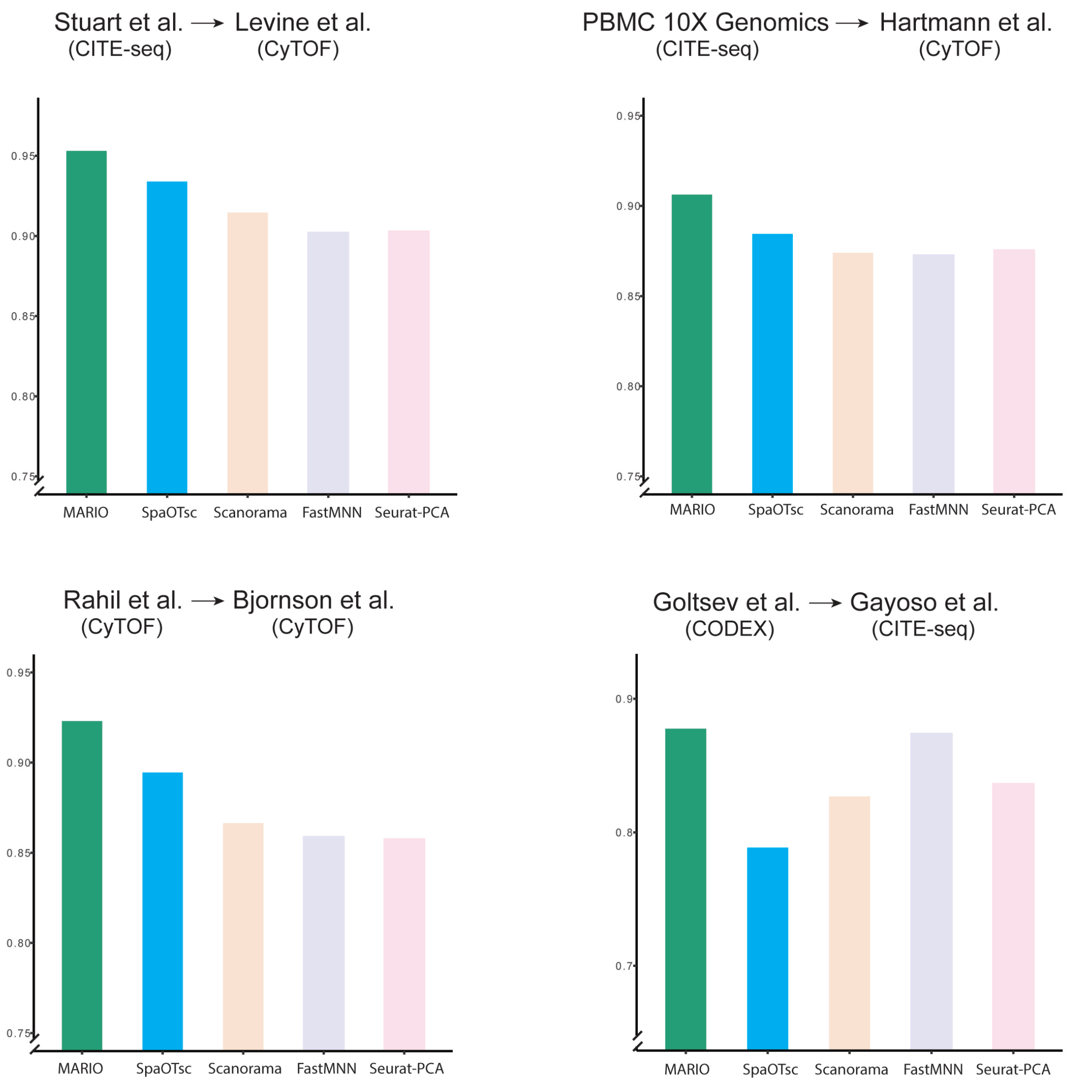

Supplementary Fig 23: MARIO matching compared to optimal transport matching Matching accuracy by MARIO and SpaOTsc (optimal transport) across different datasets presented in the manuscript.

Materials & Methods

Extensions of MARIO Pipeline.

Matching more than two datasets. Suppose we have L datasets $X_1 \in \mathbb{R}^{n_1\times(p_{\mathtt{share}}+p_1)}, \dots, X_L \in \mathbb{R}^{n_L\times(p_{\mathtt{share}}+p_L)}$. For $2 \leq \ell \leq L$, we run the usual two-dataset procedure to estimate the matching between cells in X_1 and cells in X_ℓ by $\hat{\Pi}_{1\leftrightarrow\ell}$. We then run jointly regularized filtering on each $\hat{\Pi}_{1\leftrightarrow\ell}$ separately and keep the cells in X_1 that survive all $L-1$ rounds of filtering. This gives us a cell-to-cell matching among the L datasets, from which we can construct row-wise aligned datasets $X_1^\star \in \mathbb{R}^{n\times(p_{\mathtt{share}}+p_1)}, \dots, X_L^\star \in \mathbb{R}^{n\times(p_{\mathtt{share}}+p_L)}$, where n is the number cells in X_1 that survived all $L-1$ rounds of filtering.
To jointly embed all the aligned datasets, we use generalized canonical correlation analysis (gCCA) (1). It is well known that gCCA does not admit a unique formulation (2). We take the following formulation which best suits our goal of obtaining joint embeddings:

$$\{\hat{W}_\ell\}_{\ell=1}^L = \underset{\substack{W_\ell\in\mathbb{R}^{(p_{\mathtt{share}}+p_\ell)\times r}\\ \forall 1\leq\ell\leq L}}{\operatorname{argmin}} \sum_{\ell\neq\ell'} \|X_\ell^\star W_\ell - X_{\ell'}^\star W_{\ell'}\|_F^2$$

$$\text{subject to } W_\ell^\top \hat{\Sigma}_{\ell\ell} W_\ell = I_r, \qquad \hat{\Sigma}_{\ell\ell} = \frac{(X_\ell^\star)^\top X_\ell^\star}{n},$$

where $\|\cdot\|_F$ is the Frobenius norm, $1 \leq r \leq p_{\mathtt{share}} + \min_\ell p_\ell$ is the number of components to keep, and $X_\ell \hat{W}_\ell$ is the embedding for the ℓ-th dataset.
To solve the above optimization problem, we take a block coordinate descent approach. This approach again needs preliminary estimators $\{\hat{W}_\ell^{(0)}\}$. To obtain those preliminary estimators, we first run the classical CCA on the first two datasets and obtain the projection matrices $\hat{W}_1^{(0)}, \hat{W}_2^{(0)}$, so that $X_1^\star\hat{W}_1^{(0)}$ and $X_2^\star\hat{W}_2^{(0)}$ are the sample canonical scores for $X_1^\star$ and $X_2^\star$, respectively. Then, for each $\ell \geq 3$, we run least squares regression using $(X_1^\star\hat{W}_1^{(0)} + X_2^\star\hat{W}_2^{(0)})/2$ as the response and $X_\ell^\star$ as the feature matrix. The resulting regression coefficient is then taken to be $\hat{W}_\ell^{(0)}$.
Given the preliminary estimators, we are ready to enter the block coordinate descent iteration. We first demonstrate how to solve for the first columns of $\{\hat{W}_\ell\}$. Suppose at iteration t, we are given preliminary estimators $\{\hat{w}_\ell^{(1,t)}\}$, where $\hat{w}_\ell^{(1,t)} \in \mathbb{R}^{p_{\mathtt{share}}+p_\ell}$. We then proceed as follows. For every $1 \leq \ell \leq m$, we run a least squares regression with the response being the current average scores (not counting ℓ itself), i.e., $(\sum_{\ell'<\ell} X_\ell^\star \hat{w}_{\ell'}^{(1,t+1)} + \sum_{\ell'>\ell} X_\ell^\star \hat{w}_{\ell'}^{(1,t)})/(L-1)$, and with the feature matrix being $X_\ell^\star$. Denote the resulting regression coefficient as $\tilde{w}_\ell^{(1,t+1)}$. We take $\hat{w}_\ell^{1,t+1} = \tilde{w}_\ell^{(1,t+1)}/\|\tilde{w}_\ell^{(1,t+1)}\|_2$. We run the above procedure for 500 iterations and let $\{\hat{w}_\ell^{(1,T)}\}$ be the first columns of $\{\hat{W}_\ell\}$.
We now discuss how to solve for the j-th columns of $\{\hat{W}_\ell\}$, where $j \geq 2$. We start by running a least squares regression with $X_\ell^\star$ being the response and the first $j-1$ scores of $X_\ell^\star$ (i.e., $X_\ell^\star(\hat{W}_\ell)_{\bullet,1:j-1}$, where $(\hat{W}_\ell)_{\bullet,1:j-1}$ is the first $j-1$ columns of $\hat{W}_\ell$) being the feature matrix. The residual of this regression is denoted as $\tilde{X}_\ell^\star$. Now suppose at iteration t, we are given preliminary estimators $\{\hat{w}_\ell^{(j,t)}\}$, where $\hat{w}_\ell^{(j,t)} \in \mathbb{R}^{p_{\mathtt{share}}+p_\ell}$. We proceed as follows. For every $1 \leq \ell \leq L$, we run a least squares regression with the response being $(\sum_{\ell'<\ell} \tilde{X}_\ell^\star \hat{w}_{\ell'}^{(j,t+1)} + \sum_{\ell'>\ell} \tilde{X}_\ell^\star \hat{w}_{\ell'}^{(j,t)})/(L-1)$, and with the feature matrix being $\tilde{X}_\ell^\star$. Denote the resulting regression coefficient as $\tilde{w}_\ell^{(j,t+1)}$. We then run a least squares regression with the response being $\tilde{X}_\ell^\star \tilde{w}_\ell^{(j,t+1)}/\|\tilde{w}_\ell^{(j,t+1)}\|_2$ and the feature matrix being $X_\ell^\star$. The resulting regression coefficient is taken to be $\hat{w}_\ell^{j,t+1}$. We run the above procedure for 500 iterations and let $\{\hat{w}_\ell^{(j,T)}\}$ be the j-th columns of $\{\hat{W}_\ell\}$.

Speeding up MARIO cell matching via distance sparsification. Standard implementations of the one-to-one matching run in $\mathcal{O}((n_{\mathrm{x}}+n_{\mathrm{y}})^3)$ time. However, if the distance matrix $\mathscr{D}$ is sparse (i.e., a lot of entries are infinity, meaning that such a pair is a priori infeasible), then the time complexity can further be reduced. For example, if one regards the distance matrix as a bipartite graph and let (i,j) denote an edge if $\mathscr{D}_{ij} < \infty$, then it is possible to solve the problem in $\tilde{\mathcal{O}}((n_{\mathrm{x}}+n_{\mathrm{y}})|E|)$ time, where $|E|$ is the number of edges and $\tilde{\mathcal{O}}$ hides poly-log factors (3).
A natural attempt is to manually sparsify $\mathscr{D}$ so that for each row, only $k \ll n_{\mathrm{y}}$ smallest entries are finite. Let $\mathscr{D}^{(k)}$ be the sparsified matrix. In theory, there exists a critical value of $k^\star$ such that: (1) the distance matrix $\mathscr{D}^{(k^\star)}$ can give a valid matching; and (2) if one sparsifies it further (i.e., use $\mathscr{D}^{(k)}$ for $k < k^\star$), then there is no valid matching. We give an algorithm for computing this critical value. For any fixed k, we can test if $\mathscr{D}^{(k)}$ can give a valid matching by computing the maximum-cardinality matching, which can be done in $\mathcal{O}(kn_{\mathrm{x}}\sqrt{n_{\mathrm{x}}+n_{\mathrm{y}}})$ time using the Hopcroft–Karp algorithm (4). We can then use binary search to search for the critical value $k^\star$. In the worst case (i.e., when $k^\star = n_{\mathrm{y}}$), the whole procedure runs in $\mathcal{O}(\log(n_{\mathrm{y}})n_{\mathrm{x}}n_{\mathrm{y}}\sqrt{n_{\mathrm{x}}+n_{\mathrm{y}}})$ time, which is already much faster than the $\mathcal{O}((n_{\mathrm{x}}+n_{\mathrm{y}})^3)$ time needed to compute the matching using the original distance matrix. In practice, since $k^\star$ is usually very small compared to n_{y}, the running time of the whole procedure can be even faster. This procedure generalizes the strategy taken by (5), which only works when the distance matrix is computed using a single feature.

Given the knowledge of $k^\star$, we sparsify the distance matrix with some user-specified $k \geq k^\star$ (denoted as `sparsity` in the MARIO package) and apply the LAPJVsp algorithm (an algorithm specifically designed to tackle sparse inputs) (6) to compute the matching.
In practice, we can further speed up the matching process by randomly splitting the data into n (in MARIO package denoted as `n_batch`) evenly-sized batches, computing the matching for each batch, and stitching the batch-wise matchings together.

Details on data pre-processing and analysis.

Preprocessing and analysis of human bone marrow datasets. CyTOF data measuring 32 proteins in healthy human bone marrow cells from levine et al (7)) was downloaded from GitHub https://github.com/lmweber/benchmark-data-Levine-32-dim. Cells gated as HSPCs, CD4 T cell, CD8 T cell, B cell, monocyte, NK cell and pDC from the paper were selected and a total of 102,977 cells were used. CITE-seq dataset measuring 25 proteins and RNA expression of healthy human bone marrow cells was acquired using `bmcite` in the R package `SeuratData`. Cells annotated as HSPCs, CD4 T cell, CD8 T cell, B cell, monocyte, NK cell, and pDC from the paper, comprising a total of 29,007 cells, were used. For evaluation purpose, the annotations were unified between datasets (eg. names of populations). Marker CD11c, CD123, CD14, CD16, CD19, CD3, CD34, CD38, CD4, CD45RA, CD8, and HLA-DR were shared across datasets. During matching, CITE-seq cells were used to match against CyTOF cells, where the input of CITE-seq cells were pre-normalized counts from `bmcite` and the input of CyTOF cells were values with arcsine transformation (cofactor = 5). The MARIO parameters used are `n_components_ovlp` = 10, `n_components_all` = 20, `sparsity` = 1000, `bad_prop` = 0.2, and `n_batch` = 4.
The t-SNE plots were generated using the scaled shared protein features across datasets (pre-integration) or the first 10 components for the CCA scores (MARIO integration), using the `Rtsne()` function with default settings in R package `Rtsne`. The heatmap was produced using `heatmap.2()` in the R package `gplots`, with z-scaled CITE-seq and CyTOF protein expression levels. The matched or original values of protein/RNA overlaid with t-SNE plots were generated with the function `Featureplot()` in R package `Seurat`. The detailed process of benchmarking MARIO against other methods is further described in the Benchmarking section in the Supplementary Methods section.

Preprocessing and analysis of cross species H1N1/IFN gamma challenged datasets. CyTOF data measuring 42 proteins in blood cells from humans challenged with H1N1 (8) virus was acquired from flow repository FR-FCM-Z2NZ 39. Three donors were used (id = “101”, “107”, “108”). The dataset was randomly downsampled to 120,000 cells, arcsine transformed with cofactor = 5, and subsequently clustered via the default `Seurat` clustering pipeline with all available antibody markers. Cell types were then manually annotated based on their expression profile. A total of 102,147 annotated cells were used. CyTOF data measuring 39 proteins of whole blood cells from human, rhesus macaque and cynomolgus monkey challenged with Interferon gamma (9) were acquired from flow repository FRFCM-Z2ZY 35. Three donors of each species (human: “7826”, “7718”, “2810”; rhesus macaque: “D00522”, “D06022”, “D06122”; cynomolgus monkey: “D07282”, “D07292”, “D07322”) were used. Cells gated as Erythrocytes, Platelets and CD4+CD8+ cells in the paper were excluded from downstream analysis. Each individual dataset was randomly downsampled to 120,000 cells, arcsine transformed with cofactor = 5, then clustered with `Seurat` using all the markers, followed by manually annotation and then removal of cells with ambiguous annotations. Total cell numbers for matching were 114,175 (human); 112,218 (rhesus macaque); 91,409 (cynomolgus monkey). During matching, human H1N1 challenged cells were matched against human, rhesus macaque and cynomolgus monkey IFN gamma-stimulated cells separately, and cells that matched across all four datasets were used for downstream analysis. Arcsine transformed values were used for matching. The MARIO parameters used are `n_components_ovlp` = 20, `n_components_all` = 15, `sparsity` = 1000, `bad_prop` = 0.1, and `n_batch` = 4.
The t-SNE plot was produced by the scaled shared protein features across the dataset (pre-integration) or the first 10 components of the generalized CCA scores (MARIO integration), using the `Rtsne()` function with default setting in R package `Rtsne`. For visualization purposes, cell numbers were downsampled to 20,000 each dataset (80,000 cells in total) for t-SNE visualization. Euclidean distances between matched cells were calculated based on the integrated generalized CCA scores. Accuracy for MARIO matching results among cell types was generated by 5 repeated measurements on a randomly subsampled 5000 matched cells, and the balanced accuracy was calculated with the function `confusionMatrix()` in the R package `caret`. The expression level of Ki-67, pSTAT1 and p38 overlaid on each individual dataset's t-SNE plots was produced with the function `Featureplot()` in R package `Seurat`. Violin plots were produced based on normalized (`scale()` function, within each dataset) values of Ki-67, pSTAT1, and p38 for Monocytes, CD4 T cells subpopulations with `ggplot2`.

Preprocessing and analysis of murine spleen datasets. Tiff files of CODEX multiplexed imaging data for BALBc mouse spleen, with 28 antibodies, were acquired (10) (sample ID: 'balbc-1'). Segmentation was performed with a local implementation of Mesmer (11) , with weights downloaded from: https://deepcell-data.s3-us-west-1.amazonaws.com/model-weights/Multiplex_Segmentation_20200908_2_head.h5. Inputs of segmentation were DRAQ5 (nuclear) and CD45 (membrane). Signals from the images were capped at 99.7th percentile, with prediction parameter

`model_mpp` = 0.8. Lateral spillover signals were cleaned using REDSEA (12) with the whole cell compensation flag as previously described. To clean out aggregated B220 signals in the dataset, B220 signal inside the cytoplasm (defined by 7 pixels towards the inside of the cell boundary), was removed. Afterwards, cells with DRAQ5 signal value less than 80 were removed and signals were scaled to 0-1, with percentile cutoffs of 0.5% (floor) and 99.5% (ceiling). Cells were subsequently clustered via `Seurat`, using CODEX markers: CD45, Ly6C, TCR, Ly6G, CD19, CD169, CD3, CD8a, F480, CD11c, CD27, CD31, CD4, IgM, B220, ERTR7, MHCII, CD35, CD2135, NKp46, CD1632, CD90, CD5, CD79b, IgD, CD11b, CD106. Another round of sub-clustering was then performed for dendritic cells, and macrophage populations before manual annotation of clusters. A total of 48,332 cells labeled as B cell, CD4 T cell, CD8 T cell, Dendritic cell, Macrophage, Monocyte, Neutrophil, and NK cells were used for MARIO matching. CITE-seq data 45 of murine spleen/lymph node samples from a panel of 206 antibodies were downloaded from GitHub: `https://github.com/YosefLab/totalVI_reproducibility/tree/master/data`. Only B, CD4 T cell, CD8 T cell, dendritic, macrophage, neutrophil, and NK cells originating from the spleen, a total of 7601 cells, were used. For evaluation purpose, the annotations were unified between datasets (eg. names of populations). For matching, the input of CODEX cells are post-compensated, aggregation corrected values, excluding the Ter119 red blood cell channel. CITE-seq input were the downloaded raw counts. The CITE-seq dataset was duplicated to improve the matchability, and CODEX cells subsequently matched against CITE-seq cells, with MARIO parameters: `n_components_ovlp` = 20, `n_components_all` = 15, `sparsity` = 1000, `bad_prop` = 0.05, `n_batch` = 32, `knn` = 15.
The t-SNE plots were produced using the scaled, shared protein features across datasets (pre-integration) or the first 10 components for the CCA scores (MARIO integration), using the `Rtsne()` function with default settings in R package `Rtsne`. For visualization purposes, both datasets were downsampled to 8000 matched cells from each modality (16,000 cells in total) for t-SNE plotting. Pseudo-images of the CODEX murine spleen were colored by their cell-type annotations (Cell type based on CODEX protein annotation; Label transfering from CITE-seq annotation) and matched RNA expression levels. The label transfer of CITE-seq annotation shown in the figure was done using k-NN ($k = 15$) on the MARIO distance matrix, to ensure all CODEX cells have an annotation. The RNA expression value for pseudo-imaging plotting was capped to the 80% percentile (values equal to 0 were omitted) of that gene. For gating of B cell subtypes, CODEX proteins B220, CD19, IgM, IgD, CD21/35 and MHCII were used, and manually gated in cellengine `https://cellengine.com/`. Heatmaps of matched RNA expression level of CODEX B cell subpopulations was produced via the function `DoHeatmap()` in the R package `Seurat`, with top 50 differentially expressed genes identified in each subpopulation, via the function `FindAllMarkers()` in `Seurat`.

COVID-19 human tissue specimen collection. Lung tissues from patients who succumbed to COVID-19 were obtained during autopsy at the University Hospital Basel, Switzerland. Tissues were processed as previously described (13) and collection was approved by the ethics commission of Northern Switzerland (EKNZ; study ID #2020-00969). All patients or their relatives consented to the use of tissue for research purposes. Tissue microarrays were generated from these tissue samples in-house at the University Hospital Basel, Switzerland.

Preprocessing and analysis of COVID patient macrophage datasets. CODEX on COVID-19 samples from University Hospital Basel: CODEX acquisition of the COVID-19 tissue microarrays were performed, and post-processing and cell type annotation executed as previously described (14, 15). Data from 23 COVID-19 patients (76 tissue cores; manuscript in preparation) were acquired, and a total of 62,852 macrophages that were annotated were used for MARIO matching. Processed counts of CITE-seq data acquired with a panel of 250 antibodies from bronchoalveolar lavage fluid washes from COVID-19 patients (VIB/Ghent University Hospital) was acquired from COVID-19 Cell Atlas (16). Cells from 7 COVID-19 patients (COV002; COV013; COV015; COV024; COV034; COV036; COV037) were selected, clustered, and manually annotated on a per patient level based on their protein features, using `Seurat` as previously described. A total of 16,090 macrophages were annotated and used for subsequent MARIO matching. During MARIO matching, CODEX macrophages were matched against CITE-seq macrophages, with the MARIO running parameters: `n_components_ovlp` = 25, `n_components_all` = 25, `sparsity` = 1000, `bad_prop` = 0.1, and `n_batch` = 20.
CODEX macrophages were clustered based on their matched *C1Q* mRNA expression levels (*C1QA*, *C1QB* and *C1QC*) using the function `hcut()` with k = 2 and stand = TRUE in the R package `factoextra`. Heatmaps were produced with the scaled values from CITE-seq or CODEX, via function `heatmap.2()` in R package `gplots`. Cell-cell interaction and binned anchor analysis were performed as previously described (17). In brief, for each individual *C1Q* High or Low macrophage, the Delaunay triangulation for neighboring cells (within 100µm) was calculated based on the XY position with the `deldir` R package. To establish a baseline distribution of the distances, cells were randomly assigned to existing XY positions, for 1000 permutations. The baseline distribution of the distance was then compared to the observed distances using a Wilcoxon test (two-sided). The log2 fold enrichment of observed mean over expected mean for each interaction type was plotted for interactions with a p-value < 0.05. The test results also includes the interactions in both directions (eg. Myeloid => T and T => Myeloid). For the binned anchor analysis of *C1Q* High or Low macrophages, all cells within a 100µm range were extracted and the average percentage of specific cell types in each radius bin (in 16.66um increments) were calculated and plotted. Differential expression gene analysis was performed using the function `FindMarkers()` in the R package `Seurat`. The violin plot of DE genes were created with `ggplot2`, where mRNA expression values were normalized between 0-1 for visualization purposes. GO term

analysis was conducted via the Gene Ontology tool (18, 19) (with the biological process option activated), with the input as lists of genes that were either significantly upregulated in *C1Q* High or Low macrophages. Heatmaps of the expression pattern of differentially expressed ISG genes (identified via `FindMarkers()`), filtered using a list of 628 ISGs (20, 21) with functional annotations 67 in macrophages, was plotted with the function `heatmap.2()` from the R package `gplots`. Correlations between *C1QA* macrophage percentages and neutrophil percentages were calculated with the R function `cor()` with method `spearman`.

PANINI Validation with COVID-19 Lung Tissue Samples. Protease-free combined ISH + antibody validation experiments using PANINI as previously described (17). In brief, TMA cores cut onto glass coverslips were baked at 70°C for 1hr and then transferred to 2×5 min xylene washes, followed by deparaffinization steps $2 \times 100\%$ EtOH, $2 \times 95\%$ EtOH, $1 \times 80\%$ EtOH, $1 \times 70\%$ EtOH, $3\times$ ddH2O; 3 min each. Heat induced epitope retrieval was then performed at 97°C for 10 min using the pH-9 Dako Target Retrieval Solution (Agilent, S236784-2) in a Lab Vision PT Module (Thermo Fisher Scientific). Slides were cooled to 65°C in the PT Module and then removed for equilibration to room temperature. A hydrophobic barrier was drawn around the tissue using the ImmEdge Hydrophobic Barrier pen (Vector Labs, 310018). Afterwards, endogenous peroxidase was inactivated using RNAscope Hydrogen Peroxide from the ACDBio RNAscope Multiplex Fluorescent Reagent Kit V2 (Biotechne, 323110), for 15 min at 40°C, followed by 2×2 min ddH2O washes. Coverslips were incubated overnight at 40°C (16 hrs) with RNAScope probes targeting human *C1QA* mRNA (Biotechne, 485451). Branch amplification was performed with Multiplex Amp 1, 2, 3 and HRP-C1 in the V2 kit: Amp1 30 min at 40°C, Amp2 15 min at 40°C, Amp3 30 min at 40°C, HRP-C1 15 min at 40°C, with 2×2 min $0.5\times$ RNAscope wash Buffer (Biotechne, 310091) washes between each steps. Coverslips were then incubated with TSA-Cy3 (Akoya Biosciences, NEL744001KT) in $1\times$ RNAscope TSA Buffer at a 1:50 dilution, for 15 min at room temperature in the dark, followed by 2×2 min $0.5\times$ RNAscope wash Buffer washing. The coverslips were then washed 2×5 min with $1\times$ TBS-T, then subsequently blocked in Antibody Blocking Buffer ($1\times$ TBS-T, 5% Donkey Serum, 0.1% Triton X-100, 0.05% Sodium Azide) for 1 hour. Antibody staining was next performed at 4°C overnight (16 hrs), with anti-CD15 (1:100 dilution, clone: MC480, Biolegend, 125602) and anti-CD68 (1:100 dilution, clone: D4B9C, Cell Signaling Technology, 76437T) in Antibody Dilution Buffer ($1\times$TBS-T, 3% Donkey Serum, 0.05% Sodium Azide). After staining, coverslips were washed 3×10 min with $1\times$ TBS-T, then incubated with secondary antibodies: Anti-Mouse-Cy7 (1:250, Biolegend, 405315) and Anti-Rabbit-Alexa647 (1:250, Thermo Fisher Scientific, A-21245) in Antibody Dilution Buffer for 30 min at room temperature. Coverslips were then washed 3×10 min with $1\times$ TBS-T, stained with Hoechst 33342 (1:10000 in $1\times$ TBS-T, Thermo Fisher Scientific, H3570) for 10 min at room temperature, and mounted with ProLong™ Diamond Antifade Mountant (Thermo Fisher Scientific, P36961).

Images were collected using a Keyence BZ-X710 inverted fluorescent microscope (Keyence, Inc) configured with 4 fluorescent filters (Hoechst, Cy3, Cy5 and Cy7), and a CFI Plan Apo l 20x/0.75 objective (Nikon). The Imaging setting was: 3×5 tile per tissue core, 5 Z-stacks acquired each FOV (best focused plane used), with High Resolution setting. The exposures were: 1/50s (Hoechst), 1/250s (Cy3), 1/8s (Cy5), and 6s (Cy7). Segmentation was performed with a local implementation of Mesmer (11), with weights downloaded from: `https://deepcell-data.s3-us-west-1.amazonaws.com/model-weights/Multiplex_Segmentation_20200908_2_head.h5`. Inputs of segmentation were Hoechst (nuclear) and *C1QA* + CD68 + CD15 (membrane). Signals from the images were capped at the 99.7th percentile, with prediction parameter `model_mpp` = 0.8. Features from single cells in segmented Keyence images were extracted based on the segmentation generated above, scaled by cell size, and written out as FCS files. Cells were filtered out if too large (CellSize > 500 pixels), too small (CellSize < 45 pixels) or limited in nuclear signal (Hoechst < 3500). The signal threshold of CD15, CD68 and *C1QA* positive cells were selected for each individual tissue core, and visually assessed to minimize false negative and false positive cells. Cells positive for CD68 and *C1QA* were annotated as *C1Q* High macrophages. The correlation of *C1Q* High macrophages between PANINI and CODEX experiments were calculated with the R function `cor()` with method `spearman`. For spatial correlation analysis of C1QA expression in macrophages, the tissue core was divided into 100 sub-regions (a 10×10 grid), and the number of cells or *C1QA* signal level were summed in each individual region and plotted. Correlation was calculated with function `cor()` with method `spearman`.

Simulated data benchmarking. To evaluate MARIO performance on matching high granularity ground truth cells, we simulated proteomic data with SymSim (22). We generated a dataset consisting of twenty cell types with a total of 60 features simulated, using the function `SimulateTrueCounts`. We selected parameters that were previously used to simulate epitome type datasets (23): (`ncells_total` = 40000, `min_popsize` = 1000, `i_minpop` = 2, `ngenes` = 60, `nevf` = 10, `evf_type`= "discrete", `n_de_evf` = 5, `vary` = "s", `Sigma` = 0.2, `prop_hge` = 1, `mean_hge` = 2)

In order to create simulated data coming from two different modalities, we then subsequently added batch effect to the simulated values via function `DivideBatches`: (`nbatch` = 2, `batch_effect_size` = 5)

Then cells from each batch were separated, then randomly sampled 5000 cells from batch 1 as then modality 1 dataset, 20,000 cells from batch 2 as modality 2 dataset. To mimic the antibody panel design difference, simulated features 1-20 were shared across two batches, features 21-40 only available in modality 1, and features 41-60 only available in modality 2. Cells were then matched by different methods as described in previous sections, detailed related code can be found on our GitHub repository.

Preprocessing and analysis of human PBMC datasets. CyTOF data measuring 33 proteins of PBMC from healthy human donors in Hartmann et al (24) was downloaded from flow-repository ('FR-FCM-Z249, HD06_run1'). Cells were downsampled to 50,000, clustered using `Seurat` and manually annotated, and then a total of 38,866 annotated cells were used. CITE-seq data measuring 29 proteins of health human PBMC was retrieved from 10x genomics `https://support.10xgenomics.com/single-cell-gene-expression/datasets/3.0.2/5k_pbmc_protein_v3?`. Counts were normalized via CLR normalization with `Seurat` function `Normalizedata()`, then cells were clustered based on their protein features in `Seurat`. A total of 5,241 cells were annotated and used for matching. Markers CD11b, CD127, CD14, CD16, CD19, CD25, CD27, CD3, CD4, CD45RA, CD45RO, CD56, CD8a, HLA-DR and PD-1 were shared across datasets. During matching, CITE-seq cells were used to match against CyTOF cells, where the input of CITE-seq cells were raw counts and the input of CyTOF cells were arcsine transformed with cofactor = 5. The MARIO parameters used were: `n_components_ovlp` = 10, `n_components_all` = 15, `sparsity` = 1000, `bad_prop` = 0.2, and `n_batch` = 1. Analysis was performed the same as previously described.

Preprocessing and analysis of cross species H1N1/IL-4 challenged datasets. Human H1N1 virus challenged data is the same as described in the previous section and the same set of cells were used as input to MARIO matching.

IL-4 stimulation cross-species CyTOF data is the same cross-species dataset as described in the previous section, using the same human or animal donors as described above (human: "7826", "7718", "2810"; Rhesus macaque: "D00522", "D06022", "D06122"; Cynomolgus monkey: "D07282", "D07292", "D07322"), and the whole blood cells stimulated with IL-4. Cells gated as Erythrocytes, Platelets and CD4+CD8+ cells from the paper (9) were excluded from downstream matching and analysis. Each individual dataset was randomly downsampled to 120,000 cells, arcsine transformed with cofactor = 5, and subsequently clustered with `Seurat` using all the markers, followed by manual annotation and removal of cells with ambiguous annotations. Total cell numbers for matching were 108,538 (human); 110,328 (rhesus macaque); 90,302 (cynomolgus monkey). During matching, human H1N1 challenged cells were matched against human, rhesus macaque and cynomolgus monkey IL-4 cells separately, and cells that matched to all three other datasets were used for downstream analysis. The MARIO parameters used: `n_components_ovlp` = 20, `n_components_all` = 15, `sparsity` = 1000, `bad_prop` = 0.1, `n_batch` = 4. Analysis was performed the same as previously described.

Benchmarking extensions.

MARIO parameter selection benchmarking. To evaluate the robustness of the choice of parameters used in MARIO, we benchmarked comprehensively MARIO parameters used: Number of components used during initial matching: `n_components_ovlp`; Number of components used during refined matching: `n_components_all`; Top number of canonical correlation used: `n_cancor`; Number of components used during filtering: `n_components_filter`; Proportion of bad matches assumed: `bad_prop`; During benchmarking, MARIO parameters were set to: `n_components_ovlp` = 10 (or the maximum number available); `n_components_all` = 20 (or the maximum available), `sparsity` = 5000, `bad_prop` = 0.2 , `n_batch` = 1, unless parameters were being benchmarked.

Benchmarking on time and memory usage. Time and memory usage of MARIO on the datasets presented in Figure 2, 3, 4 were evaluated, on a linux server using Intel(R) Xeon(R) CPU E5-2650 v3 @ 2.30GHz (1 CPU during the MARIO steps). The full pipeline MARIO time usage (including initial and refined matching; best interpolation finding; joint regularized filtering; CCA calculation) was measured with the default parameters, with increasing amount of cells (50,000 cell max), and ratio of X and Y set to 1:4 (e.g. at total of 20,000 cells , X has 4000 cells and Y has 16,000 cells). The MARIO matching time usage (only including intial and refined matching) was measured with the same settings, but with three different sparsity levels: (1) minimal sparsity calculated by MARIO; (2) maximal sparsity (i.e., fully dense matching without sparsification); (3) "medium" sparsity which is in the middle point between minimal and maximum. The MARIO memory usage was measured with the same settings as the time evaluation, but the maximum number was set to 100,000 cells. The peak memory usage was measured by the function profile in the python package `memory_profiler`. The influence of sparsity level used on MARIO matching accuracy was evaluated by inputting different levels (between minimal and maximal sparsity detected by MARIO). A total of 50,000 cells were used for each dataset with a ratio between X and Y being 1:4.

Benchmark distance matrix construction methods for MARIO. To evaluate the performance of MARIO with different distance matrices, we benchmarked the initial matching accuracy of MARIO, with distance matrices constructed with linear or non-linear kernels: Pearson correlation, as described in the previous section; non-linear kernels were calculated using the python package `sklearn` with default setting. For gaussian kernel, function `rbf_kernel` was used, with default setting; for Polynomial kernel, function `polynomial_kernel` was used, with default setting; for Laplacian kernel, function `laplacian_kernel` was used, with default setting; for Sigmoid kernel, function `sigmoid_kernel` was used, with default setting. Then these distance matrices were used in MARIO initial matching, and matching accuracy was evaluated.

Benchmark against optimal transport. To evaluate optimal transport matching performance on cross modality single cell proteomic datasets, we utilized SpaOTsc (25), which is initially designed to infer spatial and signaling relationships between cells from single cell transcriptomic data, using location information captured from spatial transcriptomic datasets. To adapt the method to our scenario, we switched the originally required spatial location distance matrix to a distance matrix constructed by Pearson correlations of protein features between cells within the same modality. The four input matrices used as input for function `spatial_sc` and `transport_plan` in python package `spaotsc` were:
`df_sc`: data from modality 1, with `n` row of cells, and features from modality 1. `is_dmat`: originally required spatial distance matrix, switched to a dissimilarity matrix of cells (`m x m`), calculated from 1 - Pearson correlation of protein features, where cells are from modality 2. `cost_matrix`: dissimilarity (`n x m`) between cells from modality 1 and 2, calculated with shared features between modality 1 and 2, from 1 - Pearson correlation. `sc_dmat`: dissimilarity matrix within modality 1 (`n x n`), similarly calculated as `is_dmat`. The produced `gamma` mapping matrix (`n x m`) was used to find the matching information, where for each cell in modality 1, the cell from modality 2 that has the highest gamma value was considered the match for that cell.

Reference

1. Jon R Kettenring. Canonical analysis of several sets of variables. *Biometrika*, 58(3):433–451, 1971.
2. Sheng Gao and Zongming Ma. Sparse gca and thresholded gradient descent. *arXiv preprint arXiv:2107.00371*, 2021.
3. Éva Tardos. A strongly polynomial minimum cost circulation algorithm. *Combinatorica*, 5(3):247–255, 1985.
4. John E Hopcroft and Richard M Karp. An nˆ5/2 algorithm for maximum matchings in bipartite graphs. *SIAM Journal on computing*, 2(4):225–231, 1973.
5. Ruoqi Yu, Jeffrey H Silber, Paul R Rosenbaum, et al. Matching methods for observational studies derived from large administrative databases. *Statistical Science*, 35(3):338–355, 2020.
6. Roy Jonker and Anton Volgenant. A shortest augmenting path algorithm for dense and sparse linear assignment problems. *Computing*, 38(4):325–340, 1987.
7. Jacob H Levine, Erin F Simonds, Sean C Bendall, Kara L Davis, D Amir El-ad, Michelle D Tadmor, Oren Litvin, Harris G Fienberg, Astraea Jager, Eli R Zunder, et al. Data-driven phenotypic dissection of aml reveals progenitor-like cells that correlate with prognosis. *Cell*, 162(1):184–197, 2015.
8. Zainab Rahil, Rebecca Leylek, Christian M Schürch, Han Chen, Zach Bjornson-Hooper, Shannon R Christensen, Pier Federico Gherardini, Salil S Bhate, Matthew H Spitzer, Gabriela K Fragiadakis, et al. Landscape of coordinated immune responses to h1n1 challenge in humans. *The Journal of clinical investigation*, 130(11), 2020.
9. Zachary B Bjornson-Hooper, Gabriela K Fragiadakis, Matthew H Spitzer, Deepthi Madhireddy, Dave McIlwain, and Garry P Nolan. A comprehensive atlas of immunological differences between humans, mice and non-human primates. *Frontiers in Immunology*, page 867015, 2022.
10. Yury Goltsev, Nikolay Samusik, Julia Kennedy-Darling, Salil Bhate, Matthew Hale, Gustavo Vazquez, Sarah Black, and Garry P Nolan. Deep profiling of mouse splenic architecture with codex multiplexed imaging. *Cell*, 174(4):968–981, 2018.
11. Noah F Greenwald, Geneva Miller, Erick Moen, Alex Kong, Adam Kagel, Christine Camacho Fullaway, Brianna J McIntosh, Ke Leow, Morgan Sarah Schwartz, Thomas Dougherty, et al. Whole-cell segmentation of tissue images with human-level performance using large-scale data annotation and deep learning. *bioRxiv*, 2021.
12. Yunhao Bai, Bokai Zhu, Xavier Rovira-Clave, Han Chen, Maxim Markovic, Chi Ngai Chan, Tung-Hung Su, David R McIlwain, Jacob D Estes, Leeat Keren, et al. Adjacent cell marker lateral spillover compensation and reinforcement for multiplexed images. *Frontiers in immunology*, page 2510, 2021.
13. Thomas Menter, Jasmin D Haslbauer, Ronny Nienhold, Spasenija Savic, Helmut Hopfer, Nikolaus Deigendesch, Stephan Frank, Daniel Turek, Niels Willi, Hans Pargger, et al. Postmortem examination of covid-19 patients reveals diffuse alveolar damage with severe capillary congestion and variegated findings in lungs and other organs suggesting vascular dysfunction. *Histopathology*, 77(2):198–209, 2020.
14. Sarah Black, Darci Phillips, John W Hickey, Julia Kennedy-Darling, Vishal G Venkataraaman, Nikolay Samusik, Yury Goltsev, Christian M Schürch, and Garry P Nolan. Codex multiplexed tissue imaging with dna-conjugated antibodies. *Nature Protocols*, pages 1–36, 2021.
15. Christian M Schürch, Salil S Bhate, Graham L Barlow, Darci J Phillips, Luca Noti, Inti Zlobec, Pauline Chu, Sarah Black, Janos Demeter, David R McIlwain, et al. Coordinated cellular neighborhoods orchestrate antitumoral immunity at the colorectal cancer invasive front. *Cell*, 182(5):1341–1359, 2020.
16. Chan Zuckerberg Initiative Single-Cell COVID, Esteban Ballestar, Donna L Farber, Sarah Glover, Bruce Horwitz, Kerstin Meyer, Marko Nikolić, Jose Ordovas-Montanes, Peter Sims, Alex Shalek, et al. Single cell profiling of covid-19 patients: an international data resource from multiple tissues. *MedRxiv*, 2020.
17. Sizun Jiang, Chi Ngai Chan, Xavier Rovira-Clavé, Han Chen, Yunhao Bai, Bokai Zhu, Erin McCaffrey, Noah F Greenwald, Candace Liu, Graham L Barlow, et al. Combined protein and nucleic acid imaging reveals virus-dependent b cell and macrophage immunosuppression of tissue microenvironments. *Immunity*, 2022.
18. Michael Ashburner, Catherine A Ball, Judith A Blake, David Botstein, Heather Butler, J Michael Cherry, Allan P Davis, Kara Dolinski, Selina S Dwight, Janan T Eppig, et al. Gene ontology: tool for the unification of biology. *Nature genetics*, 25(1):25–29, 2000.
19. The gene ontology resource: enriching a gold mine. *Nucleic Acids Research*, 49(D1):D325–D334, 2021.
20. Sara Mostafavi, Hideyuki Yoshida, Devapregasan Moodley, Hugo LeBoité, Katherine Rothamel, Towfique Raj, Chun Jimmie Ye, Nicolas Chevrier, Shen-Ying Zhang, Ting Feng, et al. Parsing the interferon transcriptional network and its disease associations. *Cell*, 164(3):564–578, 2016.
21. Zhuo Zhou, Lili Ren, Li Zhang, Jiaxin Zhong, Yan Xiao, Zhilong Jia, Li Guo, Jing Yang, Chun Wang, Shuai Jiang, et al. Heightened innate immune responses in the respiratory tract of covid-19 patients. *Cell host & microbe*, 27(6):883–890, 2020.
22. Xiuwei Zhang, Chenling Xu, and Nir Yosef. Simulating multiple faceted variability in single cell rna sequencing. *Nature communications*, 10(1):1–16, 2019.
23. Hani Jieun Kim, Yingxin Lin, Thomas A Geddes, Jean Yee Hwa Yang, and Pengyi Yang. Citefuse enables multi-modal analysis of cite-seq data. *Bioinformatics*, 36(14):4137–4143, 2020.
24. Felix J Hartmann, Joel Babdor, Pier Federico Gherardini, El-Ad D Amir, Kyle Jones, Bita Sahaf, Diana M Marquez, Peter Krutzik, Erika O'Donnell, Natalia Sigal, et al. Comprehensive immune monitoring of clinical trials to advance human immunotherapy. *Cell reports*, 28(3):819–831, 2019.
25. Zixuan Cang and Qing Nie. Inferring spatial and signaling relationships between cells from single cell transcriptomic data. *Nature communications*, 11(1):1–13, 2020.
