# Supplementary material for: Robust single-cell matching and multimodal analysis using shared and distinct features
Source: Nat Methods. 2023 Jan 9;20(2):304–15. doi: 10.1038/s41592-022-01709-7 (PMC9911356; doi:10.1038/s41592-022-01709-7)
Supplement: Supplementary file 2 — Reporting Summary. [file 41592_2022_1709_MOESM2_ESM.pdf]

nature research

Corresponding author(s): Sizun Jiang

Last updated by author(s): Oct 31, 2022

Reporting Summary

Nature Research wishes to improve the reproducibility of the work that we publish. This form provides structure for consistency and transparency in reporting. For further information on Nature Research policies, see our Editorial Policies and the Editorial Policy Checklist.

Statistics

For all statistical analyses, confirm that the following items are present in the figure legend, table legend, main text, or Methods section.

n/a	Confirmed	
☐	☒	The exact sample size (*n*) for each experimental group/condition, given as a discrete number and unit of measurement
☐	☒	A statement on whether measurements were taken from distinct samples or whether the same sample was measured repeatedly
☐	☒	The statistical test(s) used AND whether they are one- or two-sided<br>*Only common tests should be described solely by name; describe more complex techniques in the Methods section.*
☒	☐	A description of all covariates tested
☐	☒	A description of any assumptions or corrections, such as tests of normality and adjustment for multiple comparisons
☐	☒	A full description of the statistical parameters including central tendency (e.g. means) or other basic estimates (e.g. regression coefficient) AND variation (e.g. standard deviation) or associated estimates of uncertainty (e.g. confidence intervals)
☐	☒	For null hypothesis testing, the test statistic (e.g. *F*, *t*, *r*) with confidence intervals, effect sizes, degrees of freedom and *P* value noted<br>*Give P values as exact values whenever suitable.*
☒	☐	For Bayesian analysis, information on the choice of priors and Markov chain Monte Carlo settings
☒	☐	For hierarchical and complex designs, identification of the appropriate level for tests and full reporting of outcomes
☐	☒	Estimates of effect sizes (e.g. Cohen's *d*, Pearson's *r*), indicating how they were calculated

Our web collection on statistics for biologists contains articles on many of the points above.

Software and code

Policy information about availability of computer code

Data collection	All code used to collect and pre-process the data used in this study is deposited to : https://github.com/shuxiaoc/mario-py<br>For the PANINI experimental validation of COVID tissues presented in Figure 5, images were collected via Keyence BZ series imaging software (2018 Ver), and stitching was performed with BZ-X Analyzer (2018 Ver).
Data analysis	All code used in data analysis in this study is deposited to : https://github.com/shuxiaoc/mario-py, code related to the this round of revision is submitted.<br>R packages used:<br>Seurat 3.2.3<br>SeuratData<br>Seurat 4.1.1 (for rpca related)<br>rliger 1.0.0<br>batchelor 1.2.4<br>flowCore 1.52.1<br>Rtsne 0.16<br>dplyr 1.0.7<br>data.table 1.14.2<br>ggplot2 3.3.5<br>plyr 1.8.7<br>stats 3.6.3<br>factoextra 1.0.7<br>RColorBrewer 1.1-3<br>gplots 3.1.3<br>doSNOW 1.0.19<br>deldir 1.0-6

foreach 1.5.2
tidyverse 1.3.1
mltools 0.3.5
parallel 1.32.1
patchwork 1.1.1
Pigengene 1.12.0
matrixStats 0.62.0
ggrepel 0.9.1
reshape2 1.4.4
Symsim 0.0.0.9000
ape 5.6-2

python package used:
scanorama 1.7.2
anndata 0.8.0
scanpy 1.9.1
spaotsc 0.2
numpy 1.20.1
pandas 1.2.1
scipy 1.6.2
scikit-learn 0.23.2
matplotlib 3.3.4
seaborn 0.11.1

For manuscripts utilizing custom algorithms or software that are central to the research but not yet described in published literature, software must be made available to editors and reviewers. We strongly encourage code deposition in a community repository (e.g. GitHub). See the Nature Research guidelines for submitting code & software for further information.

Data

Policy information about availability of data

All manuscripts must include a data availability statement. This statement should provide the following information, where applicable:

- Accession codes, unique identifiers, or web links for publicly available datasets
- A list of figures that have associated raw data
- A description of any restrictions on data availability

Publicly available datasets:

Levine et al. Human BMC CYTOF: https://github.com/lmweber/benchmark-data-Levine-32-dim
Stuart et al. Human BMC CITE-seq (From R package SeuratData, "bmcite"): https://satijalab.org/seurat/articles/weighted_nearest_neighbor_analysis.html
Zainab et al. Human H1N1 challenged whole blood CYTOF: flow repository FR-FCM-Z2NZ
Bjornson et al. Human and non-human-primate whole blood CYTOF: flow repository FRFCM-Z2ZY
Goltsev et al. Murine Spleen CODEX: https://data.mendeley.com/datasets/zjnpwh8m5b/1 (Raw images per request from Nolan Lab)
Gayoso et al. Murine Spleen CITE-seq: https://github.com/YosefLab/totalVI_reproducibility/tree/master/data
COVID-19 Cell Atlas. COVID-19 patient BALF CITE-seq (VIB/Ghent): https://www.covid19cellatlas.org/index.patient.html
Hartmann et al. Human PBMC CyTOF: flow repository FR-FCM-Z249 :HD06_run1
10x Genomics. Human PBMC CITE-seq: https://support.10xgenomics.com/single-cell-gene-expression/datasets/3.0.2/5k_pbmc_protein_v3?

All data mentioned above are also available at: https://github.com/shuxiaoc/mario-py

Data generated in this study:

Goltsev et al. COVID-19 patient Lung tissue CODEX dataset: Subset of data used in this study is deposit at: https://github.com/shuxiaoc/mario-py. Separate manuscript in preparation, full dataset will release after manuscript submission.

Simulated data generated with Symsim (Zhang et al. 2019) for ground truth analysis: method and parameters described in the Material & Methods section. Code used submitted.

Figures with associated data list:
Deposited at: https://github.com/shuxiaoc/mario-py/blob/main/Manuscript_Archive_Code/data/readme.md

Field-specific reporting

Please select the one below that is the best fit for your research. If you are not sure, read the appropriate sections before making your selection.

☒ Life sciences ☐ Behavioural & social sciences ☐ Ecological, evolutionary & environmental sciences

For a reference copy of the document with all sections, see nature.com/documents/nr-reporting-summary-flat.pdf

Life sciences study design

All studies must disclose on these points even when the disclosure is negative.

Sample size	For computational benchmarking of MARIO and other methods, we selected sufficient number of cells that is manageable in terms of time and computational power for all methods, generally ranging from 5k cells to 80k cells, dependent on the scenario. We the cell numbers are sufficient here since it is the same size or larger than most of the datasets from the same modality. Additionally, the occurrence of lower frequent cell types are still sufficiently represented during testing. Further increase of the cell number for testing will not change the conclusion presented in the manuscript.<br><br>For experiments related to COVID-19 CODEX data, all tissue cores acquired from the collection site was used in this analysis.
Data exclusions	No sample was excluded in this study.
Replication	A total of 13 different datasets (6 matching & integration cases) were described in this manuscript, confirming the effectiveness of the method.
Randomization	Randomization was performed if cells were subsampled from the original dataset. For COVID-19 related experiments presented in the manuscript, no randomization was performed we do not have multiple experimental groups or conditions.
Blinding	All data presented in the manuscript was analyzed with standardized quantitative algorithms and no qualitative measurements would be affected by observer bias.<br>When conducting and performing analysis of the COVID-19 ISH validation experiments, the researcher was blinded with the tissue core information intended for validation.

Reporting for specific materials, systems and methods

We require information from authors about some types of materials, experimental systems and methods used in many studies. Here, indicate whether each material, system or method listed is relevant to your study. If you are not sure if a list item applies to your research, read the appropriate section before selecting a response.

Materials & experimental systems

n/a	Involved in the study	
☐	☒	Antibodies
☒	☐	Eukaryotic cell lines
☒	☐	Palaeontology and archaeology
☒	☐	Animals and other organisms
☐	☒	Human research participants
☒	☐	Clinical data
☒	☐	Dual use research of concern

Methods

n/a	Involved in the study	
☒	☐	ChIP-seq
☒	☐	Flow cytometry
☒	☐	MRI-based neuroimaging

Antibodies

Antibodies used	The antibody information is described in the Material and Methods section of the manuscript.<br>primary<br>anti-CD15 (1:100 dilution, clone: MC480, Biolegend, 125602)<br>anti-CD68 (1:100 dilution, clone: D4B9C, Cell Signaling Technology, 76437T)<br><br>secondary<br>Anti- Mouse-Cy7 (1:250, Biolegend, 405315)<br>Anti-Rabbit- Alexa647 (1:250, Thermo Fisher Scientific, A-21245)
Validation	All primary and secondary antibody used in this study has been validated by the manufacturer.<br><br>CD15:<br>Verified reactivity in:<br>Human, Mouse;<br>Verified application:<br>FC - Quality tested<br>IP, WB, IHC-F, IHC-P - Reported in the literature, not verified in house<br>Validation studies listed by vendor:<br>Solter D and Knowles BB. 1978. Proc. Natl. Acad. Sci. USA. 75:5565. (IHC, IP, WB)<br>Tempest N, et al. 2018. Hum Reprod. 6:e00392. PubMed<br>Wu W, et al. 2017. Sci Rep. 7:44481. PubMed<br><br>CD68:<br>Verified reactivity in:

Human;
Verified application:
IHC, IF, Flow
Validation studies listed by vendor (first two papers):
Wang, Qirui, et al. "Vascular niche IL-6 induces alternative macrophage activation in glioblastoma through HIF-2α." Nature communications 9.1 (2018): 1-15.
Mullen, Peter J., et al. "SARS-CoV-2 infection rewires host cell metabolism and is potentially susceptible to mTORC1 inhibition." Nature communications 12.1 (2021): 1-10.

Human research participants

Policy information about studies involving human research participants

Population characteristics	*Describe the covariate-relevant population characteristics of the human research participants (e.g. age, gender, genotypic information, past and current diagnosis and treatment categories). If you filled out the behavioural & social sciences study design questions and have nothing to add here, write "See above."*
Recruitment	*Describe how participants were recruited. Outline any potential self-selection bias or other biases that may be present and how these are likely to impact results.*
Ethics oversight	*Identify the organization(s) that approved the study protocol.*

Note that full information on the approval of the study protocol must also be provided in the manuscript.
